# Influence of scan body geometry on the trueness of intraoral scanning

**DOI:** 10.1038/s41405-025-00374-0

**Published:** 2025-10-18

**Authors:** Eduardo Anitua, Asier Lazcano, Beatriz Anitua, Asier Eguia, Mohammad Hamdan Alkhraisat

**Affiliations:** 1 Private Practice in Oral Implantology, Clínica Eduardo Anitua, Vitoria, Spain; 2 University Institute for Regenerative Medicine and Oral Implantology - UIRMI (UPV/EHU-Fundación Eduardo Anitua), Vitoria, Spain; 3 BTI Biotechnology Institute, Vitoria, Spain; 4 Department of Cellular Biology and Histology, Faculty of Medicine and Nursing, Universidad del País Vasco/Euskal Herriko Unibertsitaea (UPV/EHU), Leioa, Spain

**Keywords:** Dentistry, Prosthetic dentistry

## Abstract

**Purpose:**

This study aimed to evaluate the influence of scan body geometry on the trueness of intraoral scanner (IOS) acquisitions.

**Methods:**

An in vitro study was conducted using three groups of scan bodies with varying designs. Trueness was assessed by measuring deviations from a reference model obtained using a high-precision industrial scanner. Three model types were analyzed: a fully edentulous with six implants, a partially dentate with four implants, and a partially dentate with two implants.

**Results:**

In all models, the reduced-length scan body (Group 2) showed the highest trueness, with the lowest mean deviations: 87 μm (6-implant model), 104 μm (4-implant model), and 10 μm (2-implant model). The standard design (Group 1) showed moderate deviations, while the more complex design with three flat surfaces (Group 3) consistently showed the highest deviations. Shorter, simpler designs performed best across all configurations.

**Conclusions:**

Scan body geometry, particularly length and head design, plays a critical role in scanning accuracy. Simplified, shorter scan bodies enhance trueness, while greater height or geometric complexity may compromise it. These findings suggest that optimizing scan body geometry—particularly reducing height—can enhance the accuracy of digital implant impressions, with potential implications for improving reliability in implant-supported prosthetic workflows.

## Introduction

Intraoral scanners are primarily assessed based on their accuracy and ergonomics. According to ISO 5725, accuracy encompasses two fundamental components: trueness and precision. Trueness is defined as the closeness of agreement between the average value obtained from a large series of test results and an accepted reference value, while precision refers to the closeness of agreement between independent test results obtained under stipulated conditions. High accuracy requires both high trueness—minimizing systematic errors—and high precision—reducing random variability [1]. The combination of these attributes leads to greater reliability and reduced uncertainty in digital impressions. Clinically, high trueness ensures that the digital impression accurately reflects the actual intraoral situation, which is essential for the correct fit of prosthetic restorations. High precision, in turn, guarantees consistency across repeated scans, which is critical for predictable outcomes in multi-step digital workflows. Together, these attributes directly influence restoration longevity, patient comfort, and the efficiency of the restorative process.

While multiple factors such as operator skill, patient movement, and environmental conditions may affect intraoral scanning, implant-specific variables play a decisive role in digital accuracy. Operator-dependent variables include the type of intraoral scanner (IOS) used, device head size, system calibration, ambient lighting, scanning strategy, operator experience [2], and techniques such as cut-off, rescanning, and overlap management [3]. The extent, depth, and angulation of the scan also play a role [4]. Patient-related factors involve intraoral anatomy, including tooth morphology, interdental spaces, arch width, palate shape, moisture control, existing restorations, and the presence of edentulous areas [5]. In implant scanning, specific variables such as implant depth, angulation, inter-implant distance, and scan body geometry (including length and head design, in addition to material and retention system) are particularly relevant. Factors that negatively affect scanning accuracy can lead to cumulative distortion [6]. A thorough understanding of these variables is crucial to enhancing the predictability and reliability of digital implant workflows.

Recent literature has increasingly focused on the role of scan body design in optimizing the accuracy of digital implant impressions [7]. Various studies have examined how factors such as scan body material, surface treatment, geometry, and retention mechanisms affect scan data acquisition and alignment [8–11]. Historically, the first scan bodies were manufactured entirely from polymers. However, repeated clinical use often caused deformation at the screw interface, compromising stability and scan accuracy. To address this, hybrid scan bodies combining a polymer body with a titanium base were introduced. While they improved torque resistance, the need to assemble two different components introduced potential inaccuracies at the junction. More recently, fully monolithic scan bodies have been developed, aiming to reduce variability associated with multi-component designs.

Beyond material-related limitations, geometric design also poses clinical challenges. Larger or taller scan bodies frequently extend beyond the intraoral scanner’s field of view (FOV), requiring multiple scanning passes. This increases the risk of stitching artefacts when reconstructing the 3D dataset, potentially reducing trueness. Conversely, reducing height may improve accessibility and minimize cumulative error, while additional reference surfaces could facilitate alignment but also risk introducing angular inconsistencies.

Although material composition and surface treatment of scan bodies have been widely investigated, geometric aspects such as length and head design remain less explored despite their potential clinical relevance. While many commercially available scan bodies offer adequate performance in standard clinical situations, their effectiveness in challenging conditions—such as edentulous spans or highly divergent implants—remains inconsistent. Thus, the knowledge gap lies in understanding how specific geometric modifications, particularly height reduction and the addition of reference surfaces, influence trueness under different clinical scenarios. Addressing this gap is essential for improving the predictability of digital implant workflows. From a clinical perspective, clarifying these effects has direct relevance for practitioners and patients: for clinicians, it supports the selection of scan bodies that enhance workflow reliability; for patients, it contributes to prostheses with better fit, function, and durability. Furthermore, this research advances the field of digital dentistry by providing evidence-based guidelines for scan body design optimization.

The objective of this study was to assess the impact of scan body geometry (height and reference surfaces) on the trueness of intraoral digital implant impressions. Three scan body designs were evaluated: Group 1 (a commercially available scan body from BTI Biotechnology Institute, Vitoria, Spain), Group 2 (a study-specific scan body designed with identical geometry and a 50% height reduction), and Group 3 (same as Group 2 with an additional third flat surface on the head). These designs were tested across three simulated clinical scenarios: a fully edentulous arch with six implants, a partially edentulous arch with two free-end saddles (four implants), and a short-span bridge scenario (two implants). All scans were performed by experienced operators under standardized digital conditions, following ISO 12836 protocols for evaluating the accuracy of digitizing devices. The null hypothesis (H₀) proposed that no significant differences in trueness would be observed among the different scan body geometries.

The structure of this paper is as follows: the Methods section describes the design of the in vitro models and scanning protocol; the Results section presents descriptive statistical outcomes for each group and scenario; the Discussion interprets these findings in light of previous literature and clinical implications; and the Conclusions summarize the main contributions and limitations of the study.

## Materials and methods

### Reference model measurements

Reference model measurements for this study were performed using a 3D ZEISS O-INSPECT 322 multi-sensor metrology system (ZEISS Industrial Metrology; Carl Zeiss AG; Oberkochen, Germany.). This high-precision device integrates both optical and tactile measurement capabilities, making it suitable for applications demanding tight tolerances, such as in the medical, automotive, and aerospace fields. For this study, tactile probing was employed to ensure maximum accuracy. The reference scanner was used following ISO 12836 standards for evaluating the accuracy of digitizing devices, ensuring that its specified accuracy (5 μm) aligns with international guidelines.

The system uses a high-precision contact probe—typically with a ruby or ceramic spherical tip—to physically touch predefined surface points on the object. Upon contact, the system records the exact 3D coordinates by measuring probe deflection, which is then compared to the CAD model or reference geometry. To enhance accuracy, the machine compensates for minimal deflections and operates within a calibrated 3D coordinate system. This tactile method is particularly effective for assessing complex geometries, offering greater precision than optical scanning alone. The tactile measurement deviation of the contact probe is as follows:

$${Accuracy}=\left(\,1.8+\frac{L}{250}\,\right)\mu m$$

*Equation 1: Tactile measurement accuracy*Where *L* represents the length of the measured object or feature in millimeters—the distance over which the measurement is evaluated. In this study, object lengths across the seven groups ranged from 5 mm to 10.5 mm. According to the specifications of the ZEISS O-INSPECT 322, the system’s measurement accuracy for this range is between 1.82 μm and 1.842 μm, ensuring high reliability and precision within the study parameters. Three CAD-designed models—each previously fabricated via additive manufacturing—were measured: a completely edentulous model with six implants, a partially edentulous model with four implants and a partially edentulous model with two implants. Each model was scanned five times and analyzed to evaluate dimensional accuracy by comparing tactile measurements against the original CAD files, allowing for the identification of deviations and assessment of the manufacturing precision (Tables 1–3). Inter-operator variability was assessed across five operators, each with over five years of experience in dental digital workflows, showing minimal differences and confirming the reproducibility of the scanning workflow.

**Table 1:** Centers of scan body heads and spheres in the model with six implants.

Specification	1	2	3	4	5	MAX	MIN	Max. difference	Mean
**Sphere 1**									
Diameter	3.9686	3.9685	3.9685	3.9685	3.9686	3.9686	3.9685	0.0001	3.96854
Roundness	0.0283	0.0322	0.0319	0.0317	0.0311	0.0322	0.0283	0.0039	0.03104
X_Value	27.1175	27.1175	27.1175	27.1168	27.1161	27.1175	27.1161	0.0014	27.11708
Y_Value	0.0611	0.0605	0.0608	0.0604	0.0604	0.0611	0.0604	0.0007	0.06064
Z_Value	0	0	0	0	0	0	0	0	0
**Sphere 2**									
Diameter	3.9707	3.9709	3.9697	3.9696	3.97	3.9709	3.9696	0.0013	3.97018
Roundness	0.0284	0.0287	0.0268	0.027	0.0276	0.0287	0.0268	0.0019	0.0277
X_Value	3.3974	3.3979	3.3983	3.3983	3.3983	3.3983	3.3974	0.0009	3.39804
Y_Value	29.7414	29.743	29.7429	29.7437	29.7436	29.7437	29.7414	0.0023	29.74292
Z_Value	0	0	0	0	0	0	0	0	0
**Sphere 3**									
Diameter	3.9553	3.954	3.9546	3.9543	3.9551	3.9553	3.954	0.0013	3.95466
Roundness	0.0282	0.0235	0.0275	0.0226	0.0218	0.0282	0.0218	0.0064	0.02472
X_Value	−27.1289	−27.1294	−27.1298	−27.1291	−27.1284	−27.1284	−27.1298	0.0014	−27.12912
Y_Value	0.0365	0.0355	0.0353	0.035	0.035	0.0365	0.035	0.0015	0.03546
Z_Value	0	0	0	0	0	0	0	0	0
**Protocol form**									
Flatness Position 1	0.0024	0.0023	0.0023	0.0021	0.0022	0.0024	0.0021	0.0003	0.00226
Roundness Position 1	0.0018	0.0021	0.0022	0.0021	0.0021	0.0022	0.0018	0.0004	0.00206
Flatness Position 2	0.0014	0.0012	0.0012	0.0013	0.0012	0.0014	0.0012	0.0002	0.00126
Roundness Position 2	0.0013	0.0009	0.0008	0.0009	0.001	0.0013	0.0008	0.0005	0.00098
Flatness Position 3	0.0013	0.0008	0.001	0.0013	0.0011	0.0013	0.0008	0.0005	0.0011
Roundness Position 3	0.0011	0.0011	0.0008	0.0008	0.0008	0.0011	0.0008	0.0003	0.00092
Flatness Position 4	0.0013	0.0014	0.0011	0.0013	0.0012	0.0014	0.0011	0.0003	0.00126
Roundness Position 4	0.0007	0.001	0.0009	0.0009	0.0009	0.001	0.0007	0.0003	0.00088
Flatness Position 5	0.001	0.0009	0.0007	0.0007	0.0008	0.001	0.0007	0.0003	0.00082
Roundness Position 5	0.0008	0.0008	0.0008	0.0008	0.0007	0.0008	0.0007	0.0001	0.00078
Flatness Position 6	0.001	0.0007	0.0008	0.0008	0.0008	0.001	0.0007	0.0003	0.00082
Roundness Position 6	0.0019	0.0018	0.0018	0.0019	0.0019	0.0019	0.0018	0.0001	0.00186
**Position 1**									
Angle X-Z	−17.1947	−17.2046	−17.1999	−17.1968	−17.2015	−17.1947	−17.2046	0.0099	−17.1995
Angle Y-Z	−9.5488	−9.5421	−9.5279	−9.5278	−9.5295	−9.5278	−9.5488	0.021	−9.53522
X_Value	22.4765	22.48	22.4791	22.4792	22.4786	22.48	22.4765	0.0035	22.47868
Y_Value	5.6037	5.6047	5.6073	5.6062	5.6064	5.6073	5.6037	0.0036	5.60566
Z_Value	6.5626	6.5718	6.5697	6.5707	6.5709	6.5718	6.5626	0.0092	6.56914
**Position 2**									
Angle X-Z	−12.9858	−12.9887	−12.9762	−12.9753	−12.9758	−12.9753	−12.9887	0.0134	−12.98036
Angle Y-Z	−14.7155	−14.7121	−14.7087	−14.7084	−14.7104	−14.7084	−14.7155	0.0071	−14.71102
X_Value	18.5712	18.5721	18.5725	18.5729	18.5726	18.5729	18.5712	0.0017	18.57226
Y_Value	14.0719	14.0747	14.0772	14.0761	14.0759	14.0772	14.0719	0.0053	14.07516
Z_Value	7.817	7.8252	7.8215	7.8226	7.8229	7.8252	7.817	0.0082	7.82184
**Position 3**									
Angle X−Z	−11.6318	−11.6388	−11.6367	−11.6396	−11.64	−11.6318	−11.64	0.0082	−11.63738
Angle Y−Z	−14.9833	−14.9713	−14.9603	−14.9636	−14.9627	−14.9603	−14.9833	0.023	−14.96824
X_Value	10.7266	10.7263	10.7281	10.7285	10.7284	10.7285	10.7263	0.0022	10.72758
Y_Value	23.7539	23.7588	23.7614	23.7605	23.7603	23.7614	23.7539	0.0075	23.75898
Z_Value	9.2117	9.2182	9.2143	9.2156	9.216	9.2182	9.2117	0.0065	9.21516
**Position 4**									
Angle X-Z	−3.0192	−3.0337	−3.0282	−3.0278	−3.026	−3.0192	−3.0337	0.0145	−3.02698
Angle Y-Z	−4.7633	−4.753	−4.7521	−4.7548	−4.7584	−4.7521	−4.7633	0.0112	−4.75632
X_Value	−8.9291	−8.9311	−8.9295	−8.9294	−8.929	−8.929	−8.9311	0.0021	−8.92962
Y_Value	27.4042	27.4094	27.4121	27.4112	27.4111	27.4121	27.4042	0.0079	27.4096
Z_Value	9.3413	9.3447	9.3425	9.3435	9.344	9.3447	9.3413	0.0034	9.3432
**Position 5**									
Angle X-Z	−2.5407	−2.5585	−2.5445	−2.5483	−2.546	−2.5407	−2.5585	0.0178	−2.5476
Angle Y-Z	−7.0433	−7.0431	−7.044	−7.0432	−7.0477	−7.0431	−7.0477	0.0046	−7.04426
X_Value	−18.383	−18.3856	−18.3847	−18.3846	−18.384	−18.383	−18.3856	0.0026	−18.38438
Y_Value	17.276	17.278	17.2807	17.2799	17.2797	17.2807	17.276	0.0047	17.27886
Z_Value	6.433	6.4375	6.4366	6.4372	6.4376	6.4376	6.433	0.0046	6.43638
**Position 6**									
Angle X-Z	10.0377	10.0391	10.0442	10.0396	10.0419	10.0442	10.0377	0.0065	10.0405
Angle Y-Z	−9.0344	−9.0115	−9.0155	−9.0179	−9.0164	−9.0115	−9.0344	0.0229	−9.01914
X_Value	−21.6343	−21.6379	−21.6376	−21.6375	−21.6369	−21.6343	−21.6379	0.0036	−21.63684
Y_Value	6.8092	6.8112	6.8142	6.8132	6.8132	6.8142	6.8092	0.005	6.8122
Z_Value	5.533	5.5409	5.54	5.5403	5.5409	5.5409	5.533	0.0079	5.53902

**Table 2:** Centers of scan body heads and spheres in the model with four implants.

Specification	1	2	3	4	5	MAX	MIN	Max. difference	Mean
**Sphere 1**									
Diameter	1.9365	1.9364	1.937	1.9367	1.9373	1.9373	1.9364	0.0009	1.93678
Roundness	0.0337	0.0321	0.0319	0.0323	0.0321	0.0337	0.0319	0.0018	0.03242
X_Value	−18.2375	−18.2375	−18.2374	−18.2378	−18.2377	−18.2374	−18.2378	0.0004	−18.23758
Y_Value	−25.6865	−25.6868	−25.6871	−25.6869	−25.6864	−25.6864	−25.6871	0.0007	−25.68674
Z_Value	0.0005	0.0005	0.0005	0.0005	0.0005	0.0005	0.0005	0	0.0005
**Sphere 2**									
Diameter	1.9538	1.9531	1.9537	1.9532	1.9534	1.9538	1.9531	0.0007	1.95344
Roundness	0.0314	0.0311	0.0316	0.0306	0.0313	0.0316	0.0306	0.001	0.0312
X_Value	−25.2251	−25.2257	−25.2251	−25.2262	−25.2256	−25.2251	−25.2262	0.0011	−25.22554
Y_Value	20.6949	20.6954	20.6954	20.6955	20.6949	20.6955	20.6949	0.0006	20.69522
Z_Value	0.0007	0.0006	0.0007	0.0006	0.0006	0.0007	0.0006	0.0001	0.00064
**Sphere 3**									
Diameter	1.9542	1.9563	1.9549	1.9562	1.9555	1.9563	1.9542	0.0021	1.95542
Roundness	0.0246	0.0245	0.0235	0.0243	0.0237	0.0246	0.0235	0.0011	0.02412
X_Value	21.7257	21.7262	21.7255	21.727	21.7263	21.727	21.7255	0.0015	21.72614
Y_Value	2.4956	2.4954	2.4957	2.4954	2.4955	2.4957	2.4954	0.0003	2.49552
Z_Value	5.1988	5.1988	5.1988	5.1989	5.1988	5.1989	5.1988	1E−04	5.19882
**Protocol form**									
Flatness Position 1	0.0008	0.001	0.0007	0.0019	0.0007	0.0019	0.0007	0.0012	0.00102
Roundness Position 1	0.0014	0.0013	0.0014	0.0011	0.0015	0.0015	0.0011	0.0004	0.00134
Flatness Position 2	0.001	0.0011	0.0019	0.001	0.0012	0.0019	0.001	0.0009	0.00124
Roundness Position 2	0.0016	0.0014	0.0018	0.0011	0.0011	0.0018	0.0011	0.0007	0.0014
Flatness Position 3	0.0008	0.0008	0.0017	0.0009	0.0009	0.0017	0.0008	0.0009	0.00102
Roundness Position 3	0.0007	0.0005	0.0007	0.0007	0.0006	0.0007	0.0005	0.0002	0.00064
Flatness Position 4	0.0008	0.0007	0.0009	0.0008	0.0006	0.0009	0.0006	0.0003	0.00076
Roundness Position 4	0.0015	0.0012	0.0014	0.0014	0.0014	0.0015	0.0012	0.0003	0.00138
**Position 1**									
Angle X-Z	10.3313	10.3315	10.3373	10.332	10.3347	10.3373	10.3313	0.006	10.33336
Angle Y-Z	−11.6838	−11.6859	−11.6812	−11.6787	−11.6832	−11.6787	−11.6859	0.0072	−11.68256
X_Value	−11.1503	−11.1515	−11.1501	−11.1501	−11.1501	−11.1501	−11.1515	0.0014	−11.15042
Y_Value	−25.983	−25.9823	−25.9832	−25.9825	−25.9833	−25.9823	−25.9833	0.001	−25.98286
Z_Value	6.153	6.1528	6.1521	6.1548	6.153	6.1548	6.1521	0.0027	6.15314
**Position 2**									
Angle X-Z	9.1797	9.1839	9.1772	9.18	9.1822	9.1839	9.1772	0.0067	9.1806
Angle Y-Z	−7.9473	−7.9463	−7.9484	−7.9404	−7.9443	−7.9404	−7.9484	0.008	−7.94534
X_Value	−2.7138	−2.7143	−2.7134	−2.7134	−2.7136	−2.7134	−2.7143	0.0009	−2.7137
Y_Value	−22.1904	−22.1898	−22.1907	−22.1899	−22.1905	−22.1898	−22.1907	0.0009	−22.19026
Z_Value	4.6609	4.6609	4.6602	4.6624	4.661	4.6624	4.6602	0.0022	4.66108
**Position 3**									
Angle X-Z	10.5505	10.5515	10.5481	10.5513	10.5519	10.5519	10.5481	0.0038	10.55066
Angle Y-Z	4.3245	4.3248	4.3249	4.3253	4.326	4.326	4.3245	0.0015	4.3251
X_Value	−9.4173	−9.4175	−9.4171	−9.4166	−9.4173	−9.4166	−9.4175	0.0009	−9.41716
Y_Value	21.5213	21.5218	21.5219	21.5213	21.5214	21.5219	21.5213	0.0006	21.52154
Z_Value	4.9112	4.9124	4.9118	4.9108	4.9114	4.9124	4.9108	0.0016	4.91152
**Position 4**									
Angle X-Z	10.1491	10.1495	10.1472	10.1413	10.1454	10.1495	10.1413	0.0082	10.1465
Angle Y-Z	8.7744	8.7727	8.7729	8.7623	8.7726	8.7744	8.7623	0.0121	8.77098
X_Value	−18.5353	−18.535	−18.5367	−18.536	−18.5355	−18.535	−18.5367	0.0017	−18.5357
Y_Value	22.2349	22.2341	22.234	22.2342	22.2339	22.2349	22.2339	0.001	22.23422
Z_Value	4.9596	4.961	4.9624	4.9614	4.9607	4.9624	4.9596	0.0028	4.96102

**Table 3:** Centers of scan body heads and spheres in the model with two implants.

Specification	1	2	3	4	5	MAX	MIN	Max. difference	Mean
**Sphere 1**									
Diameter	1.9659	1.9659	1.9656	1.9666	1.9657	1.9666	1.9656	0.0010	1.96594
Roundness	0.0275	0.0272	0.0272	0.0255	0.0261	0.0275	0.0255	0.0020	0.0267
X_Value	−28.6551	−28.6535	−28.6538	−28.6541	−28.6527	−28.6527	−28.6551	0.0024	−28.65384
Y_Value	18.5076	18.5068	18.5066	18.5061	18.506	18.5076	18.506	0.0016	18.50662
Z_Value	20.6327	20.6327	20.6327	20.6327	20.6328	20.6328	20.6327	0.0001	20.63272
**Sphere 2**									
Diameter	1.9656	1.9653	1.9654	1.9641	1.965	1.9656	1.9641	0.0015	1.96508
Roundness	0.0301	0.03	0.0304	0.028	0.0298	0.0304	0.028	0.0024	0.02966
X_Value	19.7412	19.7402	19.7407	19.7414	19.7397	19.7414	19.7397	0.0017	19.74064
Y_Value	26.2442	26.2444	26.2447	26.2453	26.244	26.2453	26.244	0.0013	26.24452
Z_Value	21.5928	21.5927	21.5927	21.5927	21.5927	21.5928	21.5927	0.0001	21.59272
**Sphere 3**									
Diameter	1.9436	1.9435	1.9437	1.9442	1.9441	1.9442	1.9435	0.0007	1.94382
Roundness	0.0254	0.026	0.0259	0.0269	0.0267	0.0269	0.0254	0.0015	0.02618
X_Value	−3.6226	−3.6232	−3.6234	−3.6238	−3.6235	−3.6226	−3.6238	0.0012	−3.6233
Y_Value	−28.154	−28.1534	−28.1533	−28.1535	−28.1521	−28.1521	−28.154	0.0019	−28.15326
Z_Value	22.6381	22.6381	22.6381	22.6381	22.6381	22.6381	22.6381	0.0000	22.6381
**Protocol form**									
Flatness Position 1	0.001	0.001	0.001	0.0014	0.0013	0.0014	0.001	0.0004	0.00114
Roundness Position 1	0.0008	0.0008	0.001	0.001	0.001	0.001	0.0008	0.0002	0.00092
Flatness Position 2	0.001	0.0013	0.0009	0.001	0.0009	0.0013	0.0009	0.0004	0.00102
Roundness Position 2	0.0011	0.0009	0.0009	0.0009	0.0008	0.0011	0.0008	0.0003	0.00092
**Position 1**									
Angle X-Z	5.6722	5.6738	5.6741	5.674	5.674	5.6741	5.6722	0.0019	5.67362
Angle Y-Z	−3.2216	−3.221	−3.2207	−3.2146	−3.2191	−3.2146	−3.2216	0.0070	−3.2194
X_Value	17.3558	17.3557	17.3555	17.3552	17.3554	17.3558	17.3552	0.0006	17.35552
Y_Value	−8.2484	−8.2482	−8.2479	−8.2463	−8.2469	−8.2463	−8.2484	0.0021	−8.24754
Z_Value	23.1048	23.1048	23.1047	23.106	23.1063	23.1063	23.1047	0.0016	23.10532
**Position 2**									
Angle X-Z	4.6474	4.6455	4.6464	4.6509	4.6504	4.6509	4.6455	0.0054	4.64812
Angle Y-Z	−2.1552	−2.1577	−2.1552	−2.1484	−2.1549	−2.1484	−2.1577	0.0093	−2.15428
X_Value	19.657	19.6571	19.6569	19.6585	19.6585	19.6585	19.6569	0.0016	19.6576
Y_Value	−1.5084	−1.5077	−1.508	−1.5058	−1.5069	−1.5058	−1.5084	0.0026	−1.50736
Z_Value	23.3923	23.3923	23.3922	23.3926	23.3936	23.3936	23.3922	0.0014	23.3926

Commercially available scan bodies (10.5 mm Multi-Im Universal and 10 mm Multi-Im Wide; BTI Biotechnology Institute; Vitoria, Spain) were pre-attached to the reference models. These scan bodies were in place during all measurements to ensure consistent and stable probe contact with the metrology system. Their presence provided a reliable reference surface, improving the precision and repeatability of the dimensional data acquisition.

### Calculation of the implant’s geometric center

**Fig. 1:**
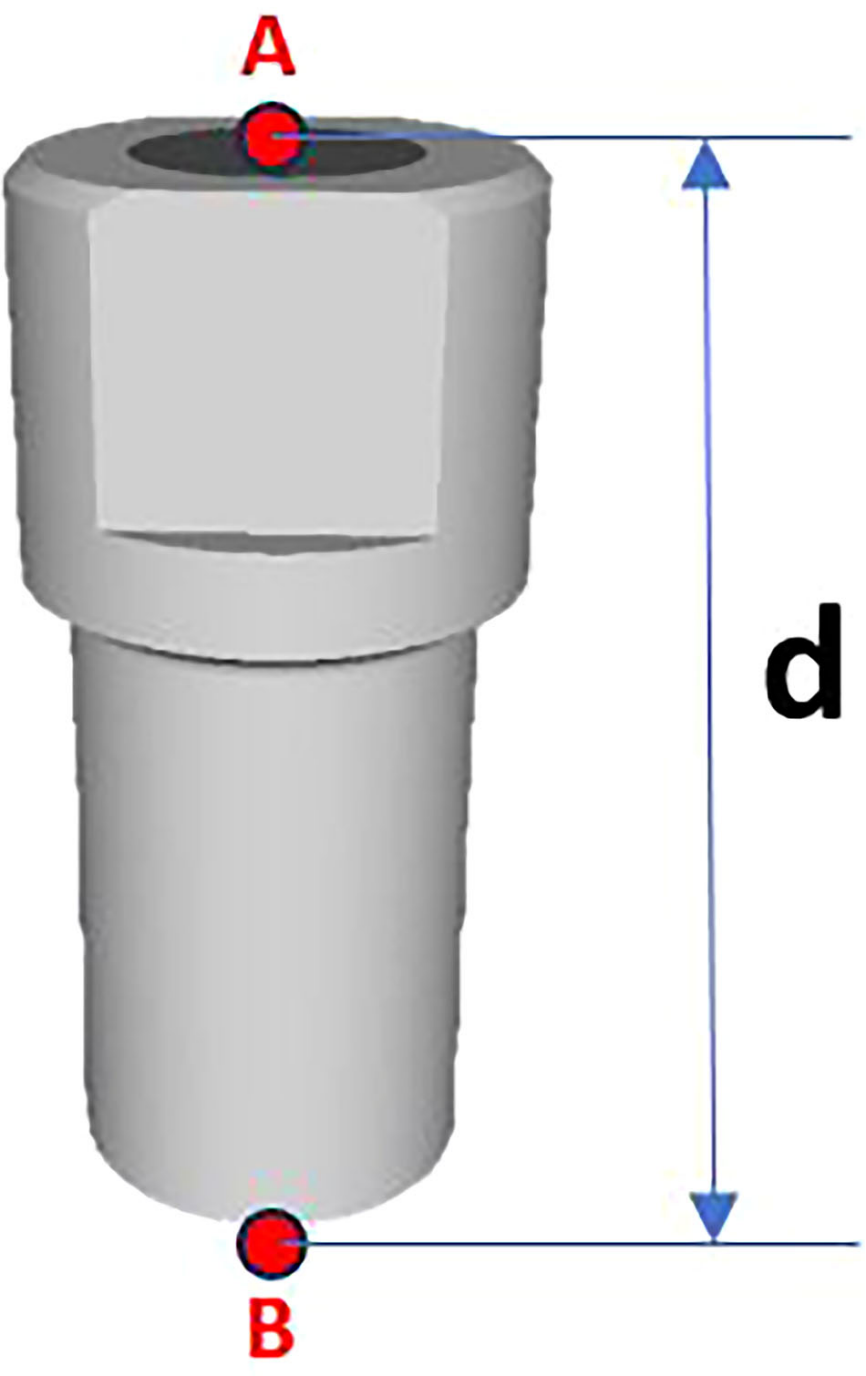
Spatial relationship betweeen the head and base of the scan body. Geometric representation showing the linear distance and spatial alignment between points A (center of the scan body head) and B(center of the scan body base).

For each model, the coordinates of the centers of the scan body heads (Point A) were determined. Based on the known lengths of the scan bodies—10.5 mm for the Multi-Im Universal and 10 mm for the Multi-Im Wide—the coordinates of Point B, located at the base of the scan body, were calculated using the following equations (Fig. 1):

$${X}_{B}={X}_{A}+\left(d\cdot \cos \left({\theta }_{{XZ}}\right)\right)\cdot \tan \left({\theta }_{{XZ}}\right)$$

*Equation 2: X coordinate value for point B*

$${Y}_{B}={Y}_{A}+\left(d\cdot \cos \left({\theta }_{{XZ}}\right)\right)\cdot \tan \left({\theta }_{{YZ}}\right)$$

*Equation 3: Y coordinate value for point B*${Z}_{B}={Z}_{A}\left(d\cdot \cos \left({\theta }_{{XZ}}\right)\right)$*Equation 4: Z coordinate value for point B*

**Fig. 2:**
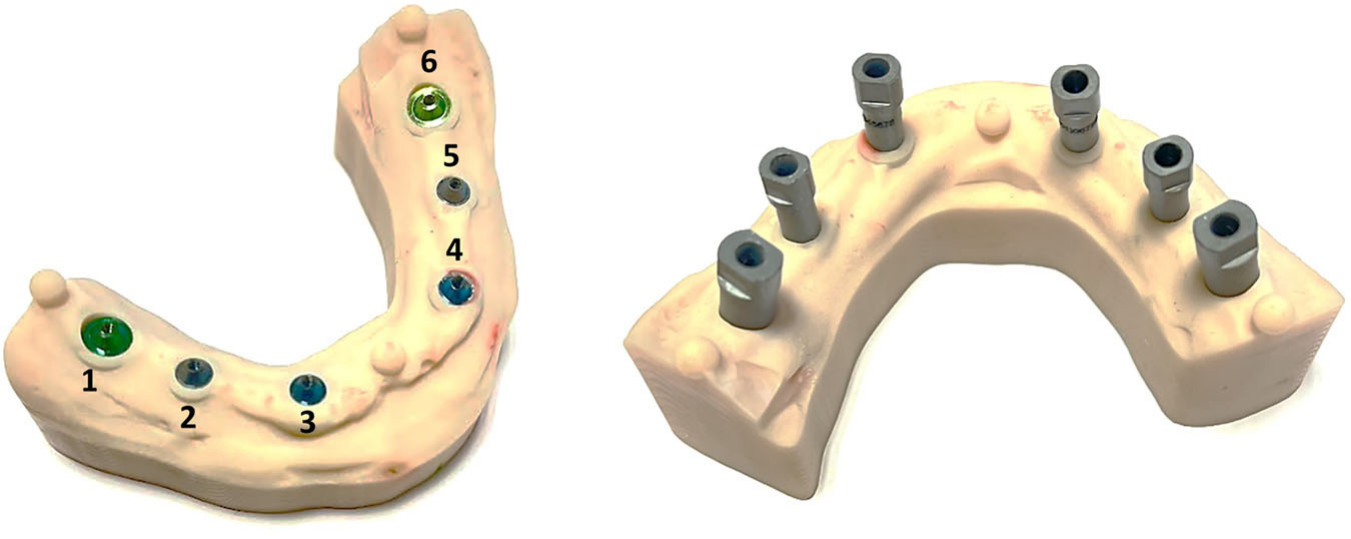
3D model of the six-implant design used in the study. The model comprises six implant analogs distributed according to the experimental configuration and three references spheres empolyed for spatial alignment and measurement calibration.

**Fig. 3:**
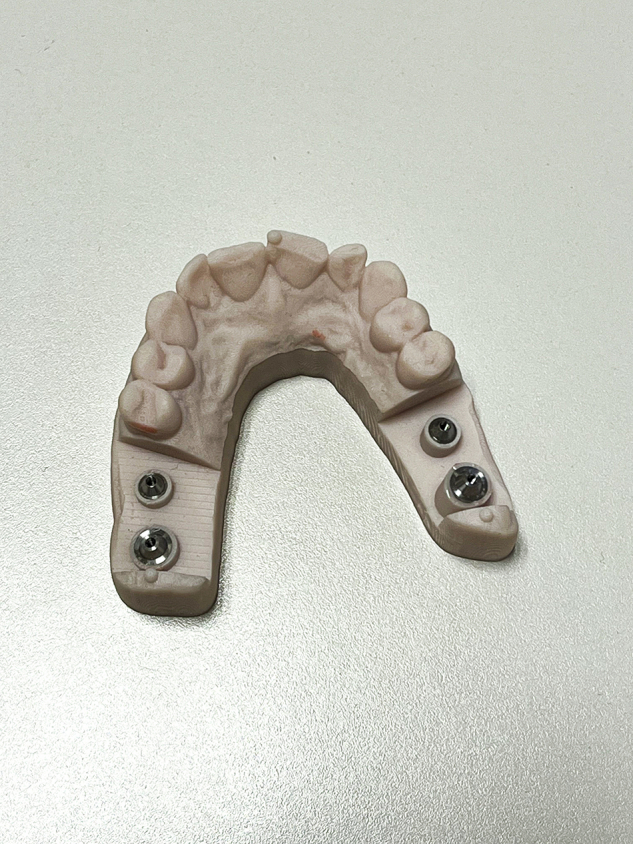
3D model of the four-implant design used in the study. The model includes four implant analogs positioned at the distal ends of each quadrant, resulting in two free-end segments. Three reference spheres were incorporated to enable spatial alignment and measurement calibration.

**Fig. 4:**
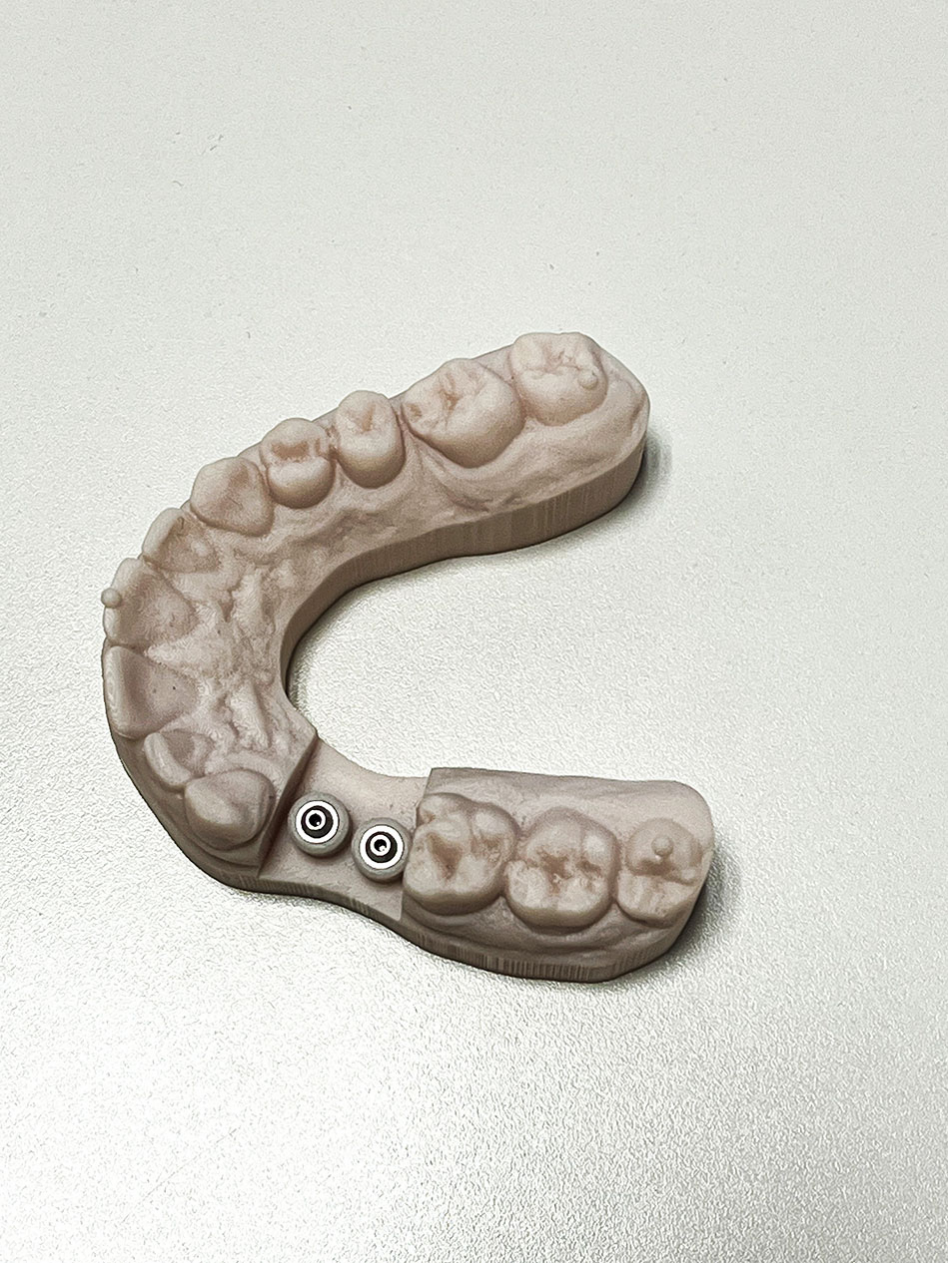
3D model of the two-implant design used in the study. The model consists of two contiguous implant analogs positiones along the dental arch. Three reference spheres were included to ensure spatial alignment and measurement calibration.

This allowed consistent definition of local coordinate systems across the three models (6-implant, 4-implant, 2-implant; Figs. 2–4). The results are presented in Tables (4–6).

**Table 4:** Calculation of base centers for scan bodies based on their length and axis position (six implant model).

Implant	X_A (mm)_	Y_A (mm)_	Z_A (mm)_	X_B (mm)_	Y_B (mm)_	Z_B (mm)_
1	22.478	5.605	6.569	25.398	7.190	−2.863
2	18.572	14.075	7.821	20.857	16.677	−2.090
3	10.727	23.758	9.215	12.776	26.418	−0.733
4	−8.92962	27.4096	9.3432	−8.377	28.279	−1.106
5	−18.384	17.278	6.436	−17.921	18.565	−3.974
6	−21.636	6.812	5.539	−23.359	8.356	−4.189

**Table 5:** Calculation of base centers for scan bodies based on their length and axis position (4 implant model).

Implant	X_A (mm)_	Y_A (mm)_	Z_A (mm)_	X_B (mm)_	Y_B (mm)_	Z_B (mm)_
1	−11.150	−25.982	6.153	−12.908	−23.989	−3.487
2	−2.713	−22.190	4.661	−4.373	−20.757	−5.607
3	−9.417	21.521	4.911	−11.334	20.742	−5.382
4	−18.535	22.234	4.961	−20.277	20.732	−4.770

**Table 6:** Calculation of base centers for scan bodies based on their length and axis position (2 implant model).

Implant	X_A (mm)_	Y_A (mm)_	Z_A (mm)_	X_B (mm)_	Y_B (mm)_	Z_B (mm)_
1	17.355	−8.247	23.105	16.319	−7.660	12.673
2	19.657	−1.507	23.392	18.807	−1.113	12.934

### 3D reconstruction of the scenario based on tactile measurements

**Fig. 5:**
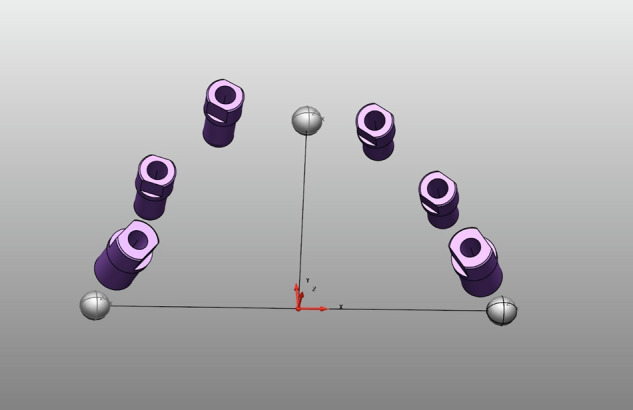
High-precision modelling of six scan bodies using Autodesk PoweShape 2024 to ensure evaluation consistency. The 3D representation illustrates the spatial distribution and alignment of the six scan bodies within the reference coordinate system, serving as the digital basis for subsequent geometric and dimensional analyses.

In Autodesk PowerShape 2024, the reference model was reconstructed from ZEISS tactile measurements by first positioning CAD spheres at the measured coordinates of the reference spheres. Implant base coordinates were then obtained as the mean of five tactile measurements per site, defining the origin point for each scan body. For orientation, the mean angular values in the X–Z and Y–Z planes were calculated, converted into directional cosines, and used to derive a unit orientation vector $\hat{v}$, which defined the Z-axis of each implant datum plane. Each plane was created with its origin at the implant base coordinates and its normal aligned with $\hat{v}$. The CAD models of the scan bodies were subsequently imported, aligned by matching their base centers to the datum origins, and oriented along the calculated vectors. This procedure was repeated for the six, four, and two-implant models, resulting in virtual assemblies that served as reference datasets for mesh-to-mesh comparison. This allowed precise measurement of deviations and standardized virtual positioning for best-fit alignment with intraoral scans. Global registration was used for overall alignment validation, but local coordinate registration was prioritized in the deviation analysis to focus on implant-specific deviation. Figure 5 illustrate the reconstruction process.

### Scan bodies

Three types of scan bodies were evaluated, all constructed from commercially pure Titanium CP Ti Grade 4.

**Fig. 6:**
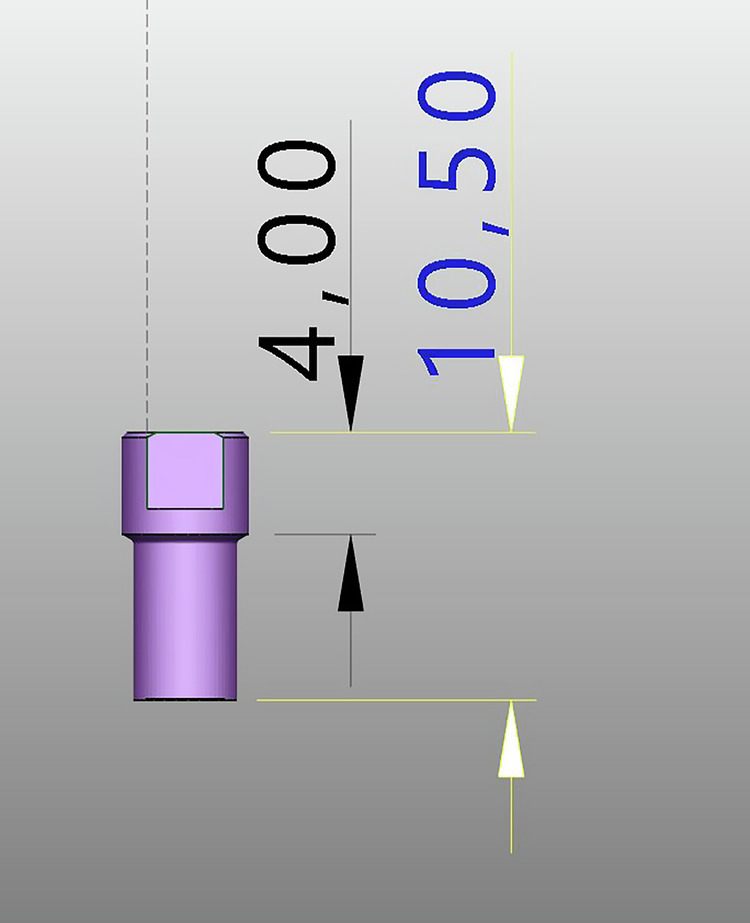
Scan body Group 1: Current design with specific length and geometric features.

**Fig. 7:**
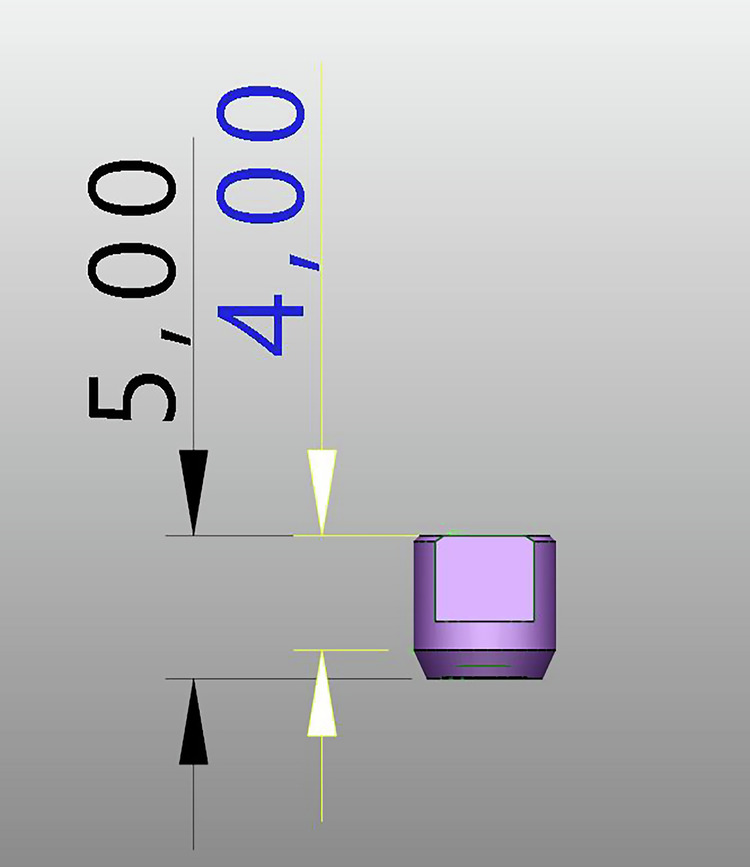
Scan body Group 2: New prototype with 50% reduced height compared to Group 1, while maintaining identical geometric and connection characteristics.

**Fig. 8:**
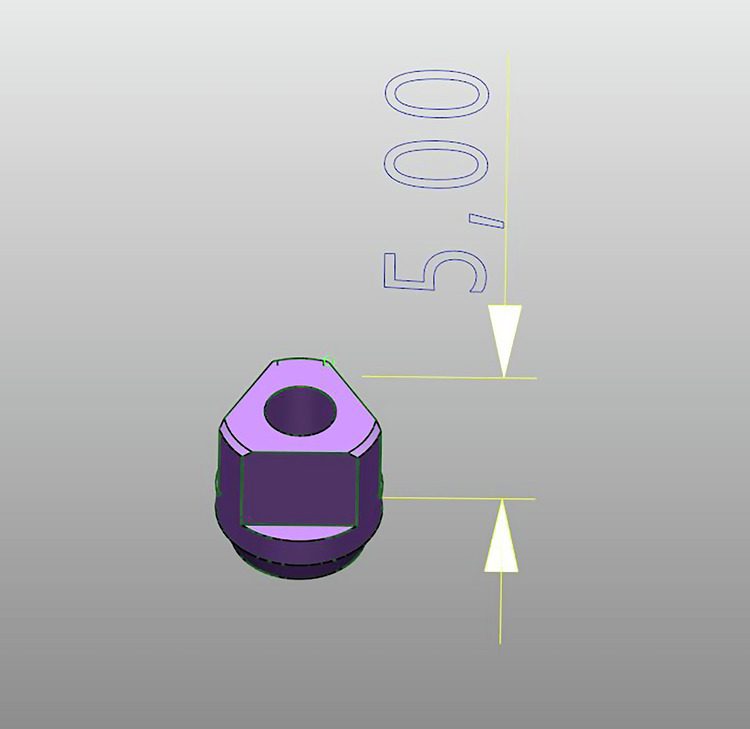
Scan body Group 3: Prototype with three flat surfaces and a length 5 mm. This design maintains the same height and connection geometry as Group 2, with the only modification being the increase from two to three flat surfaces.

- **Group 1:** Commercially available scan bodies with two flat surfaces (Fig. 6).
- **Group 2:** Experimental reduced length (5 mm), two flat surfaces (Fig. 7).
- **Group 3:** Experimental reduced length with three flat surfaces (Fig. 8).

### Data acquisition and deviation analysis

To gather data for subsequent comparisons, five experienced dentists, skilled in digital workflows and intraoral scanning, participated in the study. All scans were performed using the 3Shape TRIOS 5 intraoral scanner (IOS), following a structured protocol provided by the manufacturer and under consistent lighting conditions. The irradiance range for all scans performed was between 180 and 250 W/m², which provides a more comprehensive evaluation than illuminance measured in lux, as it considers the total radiative energy across all wavelengths, not just the visible spectrum. This ensures a more accurate assessment of environmental conditions potentially affect scan quality.

Each dentist performed three scans of each model using all three groups of scan bodies: the 6-implant model, the 4-implant model, and the 2-implant model. Each model was scanned three times with each scan body group following the manufacturer’s scanning protocol. The scanner was first positioned resting on the occlusal plane in the posterior region and kept steady for three-to-five clicks. Then, the scan proceeded slowly along the edentulous ridge, capturing the scan bodies and surrounding mucosa, and continued towards the anterior region with a steady movement to ensure accurate registration of each scan body. Once the last posterior scan body was reached, the scanner was rotated 60–90 degrees to the buccal side to perform the buccal swipe, with special attention to areas where soft tissue could interfere. Scanning then progressed along the buccal side until the opposite posterior region was reached. Finally, the scanner was rolled to the palatal/lingual side to complete the swipe, ensuring that all scan bodies were fully captured from different angles, resulting in a total of 540 scans. These scans were analyzed for deviations across the three spatial axes (X, Y, Z), producing a total of 1620 deviation analyses. This approach enabled a robust evaluation of positional accuracy across all models, scan bodies, and operators.

For each scan body in every model, a best-fit alignment was performed between the virtual scan body and the corresponding intraoral scan obtained with the IOS. This alignment ensured that the virtual scan bodies were positioned at the same coordinates as those captured during intraoral scanning, with each scan body aligned to its corresponding virtual counterpart. The Multi-IM Universal scan bodies were aligned with their respective virtual Multi-IM Universal models, while the Multi-IM Wide scan bodies were matched to their corresponding virtual Multi-IM Wide models.

This alignment process established a reliable foundation for assessing positional deviations across all scan body types, models, and operators. After aligning each scan body individually, the interior was isolated to serve as the abutment for the best-fit procedure with the previously reconstructed 3D scenario. This step ensured that the alignment accurately reflected the scan body’s position relative to the implant, enabling precise evaluation of deviations between the virtual and actual scan body placements.

### Alignment procedure

1. ***Transformation to a Common Coordinate System*** Virtual scan body meshes were translated to the same coordinate system as the meshes obtained from TRIOS 5. This was achieved by applying a 4 × 4 transformation matrix, generated through mesh-to-mesh point-based registration [11].
2. ***N-Point Registration*** Matrix values were obtained by N-point registration method, in which 6–9 five to seven geometric landmarks were manually selected on the scan bodies, specifically on the flat surfaces of the head, the top surface of the head, and at the base. These reference areas were consistently applied across all comparisons to ensure reproducibility of the alignment process. This ensured an initial alignment by re-orienting and overlapping identical regions of both meshes.
3. ***Fine Registration with Iterative Closest Point (ICP) Algorithm***

Once the initial alignment was achieved, a fine registration step was applied using the Iterative Closest Point (ICP) algorithm [12, 13]. This algorithm iteratively minimized the differences between the two sets of points, refining the alignment to achieve maximum overlap.

### Deviation calculation using Hausdorff distance

After aligning the meshes, the Hausdorff distance was calculated to quantify the dissimilarity between the 3D surfaces, capturing the maximum distance from any point on one surface to the nearest point on the other. This metric emphasizes the largest deviations, providing a rigorous and clinically relevant assessment of positional errors that could impact implant or prosthetic accuracy. In contrast, RMS or mean deviation metrics yield average values that may mask localized discrepancies. Therefore, the Hausdorff distance was chosen to ensure a robust evaluation of worst-case deviations. Given two 3D meshes M_1_ and M_2_, Haussdorf distance between them can be defined as follows:

$${Hausdorff}\left({M}_{1},{M}_{2}\right)=\max ({\max }_{{v}_{1}\in \,{M}_{1}}{\min }_{{v}_{2}\,\in \,{M}_{2}}\left|\left|{v}_{1}-{v}_{2}\right|\right|,\,{\max }_{{v}_{2}\in \,{M}_{2}}{\min }_{{v}_{1}\in \,{M}_{1}}{{\rm{||}}}{v}_{2}-{v}_{1}{{\rm{||}}})$$

*Equation 5: Hausdorff distance between two meshes M1 and M2*

For each vertex v_1_ in M_1_, the minimum distance to any vertex v_2_ in M_2_ was found and the maximum of all these minimum distances, where ||*v*_1_–*v*_2_ || denotes the Euclidean distance between vertices v_1_ and v_2_. Similarly, for each vertex ${{{\rm{v}}}}_{2}$ in ${{{\rm{M}}}}_{2}$, the minimum distance to any vertex v_1_ in ${{{\rm{M}}}}_{1}$ was found and was taken the maximum of all these minimum distances, where || v_2_ – v_1_ || denotes the Euclidean distance between vertices v_2_ and v_1_. The overall Hausdorff distance between the two 3D meshes is then the maximum of the two computed values [14, 15].

**Fig. 9:**
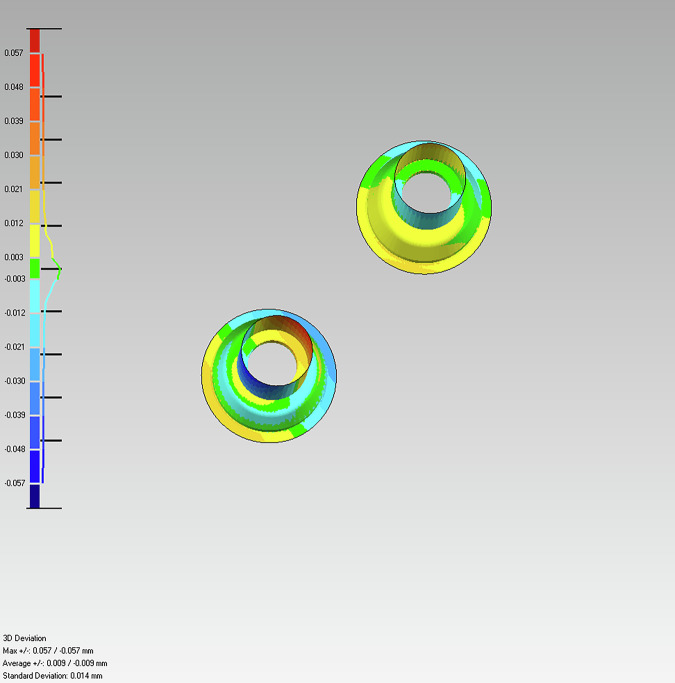
Histogram of point cloud deviation: distribution analysis for each measurement. The histogram shows the frequency of points within specific deviation ranges (in mm). The number of points corresponding to each deviation is indicated by the line adjacent to the bars, providing a quantitative overview of deviated points from the ground truth.

The resulting deviation values were visualized as *colour-coded surface maps*, where red-yellow indicated positive dimensional differences, green indicated no significant deviation, and blue indicated negative differences (Fig. 9). Histograms were generated for each analysis to represent the distribution of all mesh points.

After best-fit alignment, the abutment contour diameter was individualized, and a local coordinate system was defined at the center of each abutment. The distance between this local system and the original reference system was calculated as:

$$d\left(o,f\right)=\sqrt{{({X}_{o}-{X}_{f})}^{2}+{({Y}_{o}-{Y}_{f})}^{2}+{({Z}_{o}-{Z}_{f})}^{2}}$$

*Equation 6: Distance between two different coordinate systems*

**Fig. 10:**
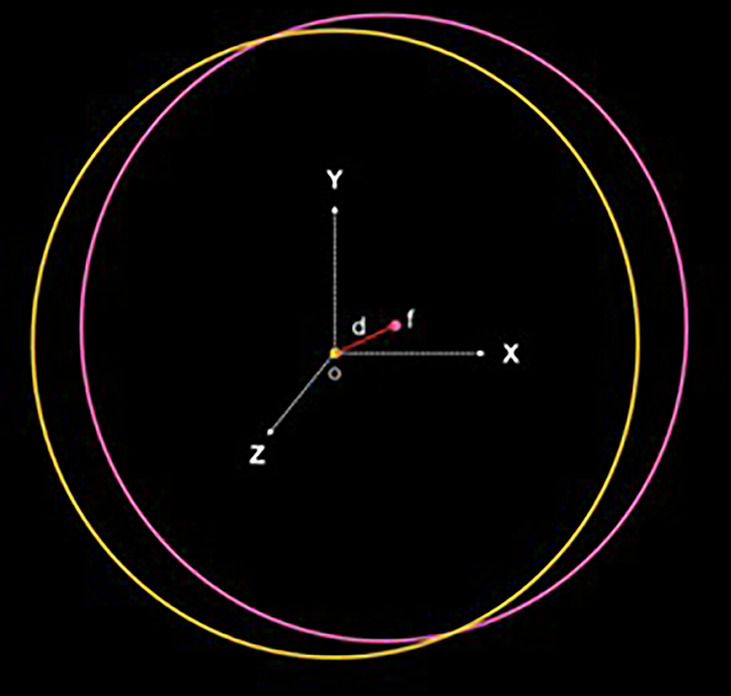
Distance between the original Coordinate System (O) and the local coordinate System (F) for each abutment. The graph illustrates the spatial displacement of each deviated abutment's local system relative to the reference global system (ground truth).

Where o represents the original coordinate system and f the new local coordinate system of each abutment (Fig. 10). Statistical analysis was performed using one-way and two-way ANOVA to assess the effects of implant number, scan body design, and operator on trueness, followed by Tukey’s HSD post-hoc tests for pairwise comparisons, with the significance level set at *p* < 0.05.

Ethical approval and consent was not applicable, as this study did not involve human participants or identifiable personal data.

## Results

Deviations were calculated separately for each implant model and operator. Each operator performed three scans per model (6, 4, and 2-implant), and the 3D Euclidean deviation was calculated from X, Y, and Z components, yielding a single deviation value for each scan.

### 6-implant model

**Fig. 11:**
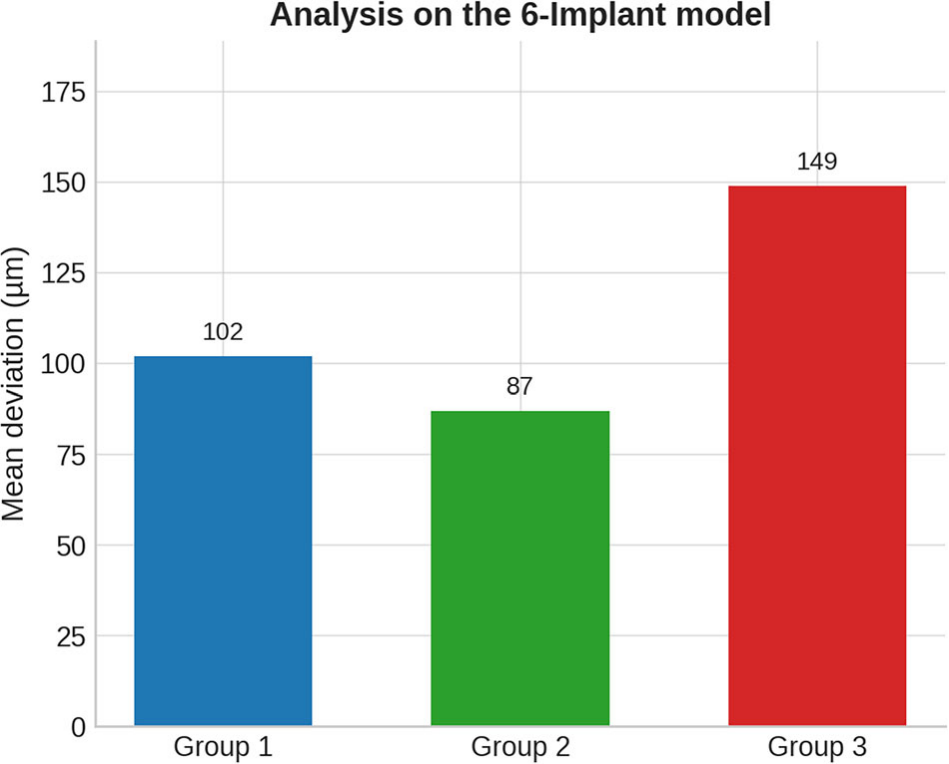
Mean deviation (µm) comparison between Group 1, Group 2, and Group 3 in the 6-implant model. The graph shows the average deviations measured for each scan body group: Group 1-102 microns, Group 2-87 microns and Group 3-149 microns.

The 6-implant model showed clear differences across groups (Fig. 11, Table 7).

- Group 1: Mean deviation 102 μm.
- Group 2: Mean deviation 87 μm, demonstrating improved trueness with reduced length.
- Group 3: Mean deviation 149 μm, where the third flat surface introduced higher deviations.

**Table 7:** Results of the analysis on the 6-implant model.

USER	TIME	Mean deviation (mm)	SD (mm)	Max + (mm)	Min- (mm)	Total deviation implant center Implant 1 (mm)	X (mm)	Y (mm)	Z (mm)	Total deviation implant center Implant 2 (mm)	X (mm)	Y (mm)	Z (mm)	Total deviation implant center Implant 3 (mm)	X (mm)	Y (mm)	Z (mm)	Total deviation implant center Implant 4 (mm)	X (mm)	Y (mm)	Z (mm)	Total deviation implant center Implant 5 (mm)	X (mm)	Y (mm)	Z (mm)	Total deviation implant center Implant 6 (mm)	X (mm)	Y (mm)	Z (mm)	Scanbody	Mean (mm)
1	A	−0.003	0.067	0.182	−0.18	**0.141**	0.055	0.129	0.003	**0.065**	0.019	0.028	0.054	**0.093**	0.081	0.004	0.044	**0.117**	0.06	0.085	0.052	**0.137**	0.103	0.087	0.023	**0.174**	0.174	0.007	0.007	**GROUP 1**	**0.12116667**
1	B	−0.001	0.039	0.148	−0.148	**0.138**	0.006	0.008	0.138	**0.125**	0.123	0.012	0.018	**0.055**	0.023	0.048	0.011	**0.031**	0.022	0.007	0.02	**0.052**	0.03	0.026	0.033	**0.056**	0.053	0.002	0.015	**GROUP 1**	**0.07616667**
1	C	0	0.055	0.219	−0.217	**0.146**	0.035	0.003	0.142	**0.041**	0.022	0.028	0.019	**0.082**	0.081	0.012	0.008	**0.081**	0.051	0.055	0.031	**0.04**	0.001	0.037	0.014	**0.035**	0	0.002	0.035	**GROUP 1**	**0.07083333**
1	A	−0.001	0.047	0.136	−0.13	**0.061**	0.054	0.007	0.026	**0.068**	0.004	0.068	0.005	**0.091**	0.007	0.065	0.063	**0.133**	0.089	0.079	0.059	**0.038**	0.003	0.037	0.008	**0.059**	0.042	0.04	0.008	**GROUP 2**	**0.075**
1	B	−0.002	0.059	0.148	−0.147	**0.146**	0.102	0.104	0.009	**0.177**	0.036	0.169	0.04	**0.045**	0.025	0.037	0	**0.128**	0.113	0.057	0.014	**0.045**	0.034	0.028	0.007	**0.056**	0.012	0.054	0.01	**GROUP 2**	**0.0995**
1	C	−0.001	0.055	0.139	−0.14	**0.009**	0.009	0	0	**0.027**	0.008	0.002	0.026	**0.157**	0.006	0.123	0.097	**0.257**	0.081	0.137	0.201	**0.201**	0.015	0.112	0.166	**0.137**	0.067	0.116	0.023	**GROUP 2**	**0.13133333**
1	A	−0.001	0.041	0.112	−0.112	**0.025**	0.013	0.01	0.019	**0.068**	0.067	0.01	0.006	**0.081**	0.064	0.047	0.014	**0.1**	0.072	0.046	0.052	**0.031**	0.006	0.024	0.018	**0.078**	0.072	0.028	0.013	**GROUP 3**	**0.06383333**
1	B	−0.001	0.057	0.169	−0.162	**0.085**	0.064	0.021	0.05	**0.12**	0.112	0.041	0.016	**0.042**	0.018	0.037	0.007	**0.137**	0.125	0.053	0.019	**0.04**	0.034	0.011	0.016	**0.052**	0.025	0.041	0.019	**GROUP 3**	**0.07933333**
1	C	0	0.043	0.125	−0.137	**0.027**	0.002	0.019	0.018	**0.037**	0.026	0.013	0.021	**0.129**	0.104	0.069	0.032	**0.098**	0.096	0.013	0.008	**0.084**	0.078	0.007	0.031	**0.166**	0.064	0.024	0.151	**GROUP 3**	**0.09016667**
2	A	−0.003	0.07	0.206	−0.207	**0.146**	0.04	0.125	0.064	**0.027**	0.008	0.023	0.01	**0.022**	0.015	0.007	0.013	**0.128**	0.014	0.054	0.115	**0.153**	0.137	0.056	0.039	**0.274**	0.187	0.061	0.191	**GROUP 1**	**0.125**
2	B	−0.003	0.071	0.206	−0.206	**0.166**	0.05	0.148	0.053	**0.041**	0.009	0.035	0.02	**0.037**	0.024	0.025	0.013	**0.039**	0.017	0.026	0.024	**0.164**	0.102	0.032	0.124	**0.369**	0.14	0.032	0.34	**GROUP 1**	**0.136**
2	C	−0.002	0.072	0.211	−0.211	**0.557**	0.077	0.313	0.453	**0.49**	0.09	0.148	0.458	**0.214**	0.015	0.041	0.21	**0.072**	0.067	0.01	0.022	**0.021**	0.007	0.009	0.018	**0.047**	0.028	0.036	0.011	**GROUP 1**	**0.2335**
2	A	−0.001	0.047	0.165	−0.145	**0.15**	0.086	0.111	0.054	**0.022**	0.006	0.019	0.007	**0.111**	0.081	0.026	0.071	**0.017**	0.009	0.01	0.01	**0.036**	0.017	0.031	0.006	**0.061**	0.06	0.005	0.009	**GROUP 2**	**0.06616667**
2	B	−0.001	0.045	0.134	−0.124	**0.146**	0.102	0.104	0.009	**0.177**	0.036	0.169	0.04	**0.045**	0.025	0.037	0	**0.128**	0.113	0.057	0.014	**0.045**	0.034	0.028	0.007	**0.056**	0.012	0.054	0.01	**GROUP 2**	**0.0995**
2	C	−0.001	0.031	0.101	−0.099	**0.154**	0.026	0.035	0.147	**0.011**	0.005	0.005	0.008	**0.037**	0.031	0.021	0.002	**0.024**	0.015	0.012	0.014	**0.026**	0.014	0.007	0.021	**0.026**	0.001	0.015	0.021	**GROUP 2**	**0.04633333**
2	A	0	0.051	0.129	−0.13	**0.087**	0.018	0.068	0.05	**0.081**	0.034	0.069	0.025	**0.052**	0.023	0.047	0.002	**0.027**	0.003	0.025	0.01	**0.034**	0.009	0.008	0.032	**0.111**	0.103	0.03	0.028	**GROUP 3**	**0.06533333**
2	B	0	0.034	0.111	−0.111	**1.518**	0.082	0.175	1.506	**0.728**	0.065	0.218	0.692	**0.361**	0.113	0.342	0.026	**0.497**	0.2	0.446	0.093	**1.432**	0.22	0.672	1.245	**2.35**	0.054	0.657	2.256	**GROUP 3**	**1.14766667**
2	C	−0.002	0.067	0.22	−0.22	**0.008**	0	0	0.008	**0.076**	0.022	0.033	0.064	**0.056**	0.049	0.025	0.008	**0.045**	0	0.045	0.005	**0.11**	0.02	0.085	0.067	**0.216**	0.056	0.196	0.072	**GROUP 3**	**0.08516667**
3	A	−0.002	0.072	0.253	−0.254	**0.069**	−0.003	−0.022	−0.065	**0.073**	0.027	0.04	0.055	**0.025**	0.007	0.013	0.02	**0.083**	−0.054	0.062	−0.01	**0.095**	0.061	−0.044	−0.057	**0.088**	0.058	0.032	0.057	**GROUP 1**	**0.07216667**
3	B	−0.001	0.044	0.156	−0.154	**0.122**	0.043	0.033	0.109	**0.025**	0.006	0.012	0.021	**0.082**	0.043	0.062	0.032	**0.076**	0.047	0.058	0.013	**0.082**	0.052	0.059	0.023	**0.074**	0.068	0.026	0.013	**GROUP 1**	**0.07683333**
3	C	−0.002	0.062	0.181	−0.187	**0.039**	0.02	0.03	0.016	**0.111**	0.005	0.061	0.093	**0.154**	0.143	0.025	0.052	**0.18**	0.168	0.059	0.028	**0.035**	0.026	0.021	0.007	**0.059**	0.047	0.033	0.01	**GROUP 1**	**0.09633333**
3	A	−0.001	0.033	0.087	−0.087	**0.067**	0.053	0.027	0.03	**0.05**	0.014	0.012	0.045	**0.048**	0.001	0.045	0.015	**0.068**	0.059	0.029	0.018	**0.041**	0.011	0.03	0.024	**0.075**	0.035	0.057	0.033	**GROUP 2**	**0.05816667**
3	B	−0.001	0.061	0.149	−0.151	**0.207**	0.193	0.073	0.001	**0.207**	0.121	0.151	0.071	**0.027**	0.018	0.019	0.006	**0.095**	0.075	0.056	0.011	**0.039**	0.005	0.029	0.025	**0.098**	0.091	0.032	0.018	**GROUP 2**	**0.11216667**
3	C	−0.001	0.07	0.164	−0.164	**0.135**	0.006	0.095	0.096	**0.194**	0.085	0.145	0.095	**0.118**	0.116	0	0.02	**0.174**	0.149	0.086	0.023	**0.042**	0.035	0.017	0.015	**0.055**	0.054	0	0.005	**GROUP 2**	**0.11966667**
3	A	0	0.042	0.132	−0.13	**0.065**	0.059	0.003	0.025	**0.099**	0.075	0.006	0.063	**0.079**	0.05	0.043	0.043	**0.101**	0.082	0.057	0.012	**0.091**	0.04	0.032	0.074	**0.015**	0.013	0.007	0	**GROUP 3**	**0.075**
3	B	−0.001	0.045	0.124	−0.12	**0.073**	0.065	0.013	0.031	**0.099**	0.07	0.035	0.061	**0.043**	0.033	0.021	0.018	**0.168**	0.088	0.076	0.121	**0.117**	0.034	0.031	0.107	**0.007**	0	0	0.007	**GROUP 3**	**0.0845**
3	C	−0.001	0.041	0.12	−0.118	**0.057**	0.03	0.038	0.03	**0.033**	0.003	0.002	0.033	**0.092**	0.076	0.048	0.015	**0.135**	0.128	0.043	0.004	**0.078**	0.046	0.043	0.045	**0.023**	0.01	0.019	0.007	**GROUP 3**	**0.06966667**
4	A	−0.002	0.068	0.239	−0.238	**0.068**	0.009	0.056	0.037	**0.049**	0.038	0.021	0.021	**0.114**	0.101	0.052	0.001	**0.049**	0.021	0.022	0.037	**0.019**	0.002	0.019	0.004	**0.036**	0.035	0.007	0.005	**GROUP 1**	**0.05583333**
4	B	−0.003	0.077	0.214	−0.214	**0.045**	0.033	0.027	0.013	**0.105**	0.048	0.008	0.092	**0.213**	0.199	0.073	0.018	**0.329**	0.316	0.076	0.044	**0.197**	0.186	0.004	0.064	**0.036**	0.021	0.026	0.013	**GROUP 1**	**0.15416667**
4	C	−0.003	0.085	0.262	−0.26	**0.262**	0.18	0.189	0.017	**0.146**	0.125	0.067	0.032	**0.03**	0.027	0.006	0.013	**0.149**	0.119	0.073	0.049	**0.053**	0.04	0.034	0.011	**0.027**	0.003	0.022	0.014	**GROUP 1**	**0.11116667**
4	A	−0.003	0.066	0.168	−0.16	**0.012**	0.01	0.004	0.005	**0.16**	0.122	0.085	0.059	**0.165**	0.16	0.022	0.035	**0.199**	0.176	0.094	0.001	**0.021**	0.013	0.012	0.009	**0.094**	0.032	0.087	0.015	**GROUP 2**	**0.1085**
4	B	−0.003	0.051	0.157	−0.157	**0.122**	0.105	0.061	0.008	**0.039**	0.009	0.01	0.036	**0.115**	0.03	0.107	0.032	**0.121**	0.105	0.061	0.003	**0.012**	0.009	0.008	0	**0.073**	0.002	0.072	0.015	**GROUP 2**	**0.08033333**
4	C	−0.002	0.043	0.125	−0.123	**0.096**	0.062	0.071	0.019	**0.107**	0.025	0.017	0.103	**0.073**	0.02	0.002	0.07	**0.144**	0.139	0.011	0.037	**0.038**	0.031	0.015	0.016	**0.032**	0.027	0.016	0.005	**GROUP 2**	**0.08166667**
4	A	−0.001	0.035	0.105	−0.097	**0.002**	0.002	0	0	**0.087**	0.017	0.017	0.083	**0.114**	0.107	0.02	0.031	**0.116**	0.112	0.029	0.004	**0.087**	0.039	0.031	0.071	**0.016**	0.006	0.014	0	**GROUP 3**	**0.07033333**
4	B	0	0.065	0.182	−0.182	**0.317**	0.067	0.283	0.125	**0.138**	0.044	0.13	0.012	**0.021**	0.001	0.001	0.021	**0.082**	0.018	0.017	0.078	**0.015**	0	0.002	0.015	**0.006**	0.006	0	0	**GROUP 3**	**0.0965**
4	C	−0.004	0.067	0.185	−0.186	**0.098**	0.036	0.083	0.036	**0.039**	0.027	0.009	0.027	**0.113**	0.106	0.012	0.036	**0.136**	0.105	0.083	0.024	**0.046**	0.035	0.028	0.005	**0.202**	0.195	0.053	0.001	**GROUP 3**	**0.10566667**
5	A	−0.002	0.081	0.339	−0.329	**0.018**	0.016	0.006	0.002	**0.092**	0.024	0.017	0.087	**0.023**	0.014	0.006	0.017	**0.118**	0.002	0.057	0.103	**0.022**	0.016	0.005	0.014	**0.039**	0.031	0.023	0.006	**GROUP 1**	**0.052**
5	B	−0.003	0.082	0.362	−0.359	**0.135**	0.012	0.067	0.117	**0.032**	0.008	0.03	0.009	**0.019**	0.011	0.011	0.009	**0.056**	0.043	0.006	0.034	**0.085**	0.043	0.072	0.011	**0.022**	0.004	0.02	0.007	**GROUP 1**	**0.05816667**
5	C	−0.002	0.061	0.271	−0.269	**0.13**	0.059	0.112	0.026	**0.129**	0.06	0.034	0.109	**0.113**	0.041	0.029	0.101	**0.086**	0.061	0.055	0.025	**0.021**	0.016	0.014	0.001	**0.087**	0.013	0.086	0.003	**GROUP 1**	**0.09433333**
5	A	−0.001	0.063	0.237	−0.235	**0.189**	0.007	0.182	0.049	**0.142**	0.015	0.134	0.043	**0.024**	0.01	0.022	0.001	**0.062**	0.058	0.018	0.01	**0.026**	0.011	0.016	0.018	**0.021**	0.016	0.001	0.013	**GROUP 2**	**0.07333333**
5	B	−0.001	0.052	0.174	−0.17	**0.029**	0.025	0.013	0.005	**0.061**	0.015	0.016	0.057	**0.109**	0.01	0.109	0	**0.127**	0.018	0.123	0.021	**0.099**	0.026	0.095	0.007	**0.139**	0.069	0.111	0.047	**GROUP 2**	**0.094**
5	C	0	0.056	0.188	−0.187	**0.098**	0.019	0.089	0.036	**0.08**	0.004	0.076	0.023	**0.033**	0.011	0.02	0.024	**0.031**	0.025	0.009	0.015	**0.049**	0.03	0.037	0.012	**0.093**	0.056	0.074	0.012	**GROUP 2**	**0.064**
5	A	−0.001	0.039	0.124	−0.117	**0.024**	0.021	0.011	0	**0.08**	0.014	0.038	0.069	**0.043**	0.023	0.005	0.036	**0.125**	0.057	0.111	0.002	**0.047**	0.008	0.002	0.046	**0.014**	0.006	0.012	0.003	**GROUP 3**	**0.0555**
5	B	−0.001	0.054	0.173	−0.168	**0.122**	0.109	0.022	0.051	**0.117**	0.095	0.066	0.007	**0.057**	0.037	0.001	0.043	**0.095**	0.039	0.083	0.021	**0.011**	0.008	0.007	0.002	**0.019**	0.014	0.011	0.002	**GROUP 3**	**0.07016667**
5	C	−0.001	0.058	0.206	−0.205	**0.182**	0.063	0.147	0.088	**0.119**	0.046	0.109	0.008	**0.03**	0.019	0.017	0.016	**0.105**	0.036	0.099	0.003	**0.037**	0.018	0.031	0.009	**0.014**	0	0.008	0.011	**GROUP 3**	**0.08116667**

### 4-implant model

**Fig. 12:**
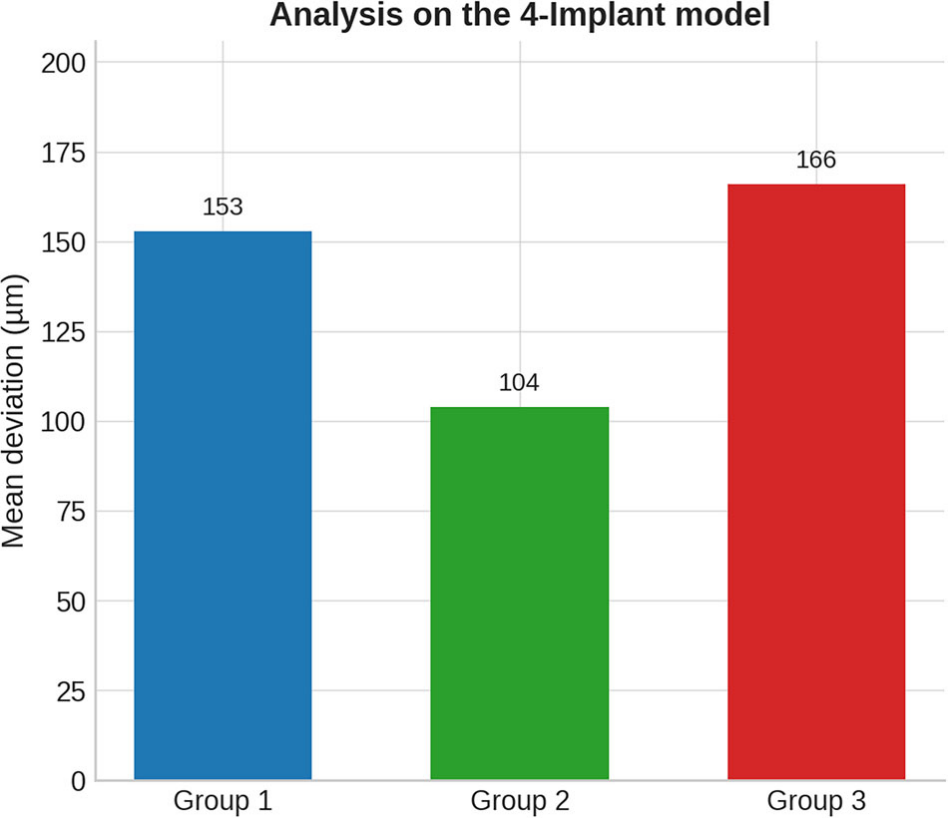
Mean deviation (µm) comparison between Group 1, Group 2, and Group 3 in the 4-implant model. The graph shows the average deviations measured for each scan body group: Group 1-153 microns, Group 2-104 microns and Group 3-166 microns.

The 4-implant configuration also revealed design-dependent variability (Fig. 12. Table 8).

- Group 1: Mean deviation 153 μm.
- Group 2: Mean deviation 104 μm, again showing better trueness with reduced length.
- Group 3: Mean deviation 166 μm, the least accurate in this configuration.

**Table 8:** Results of the analysis on the 4-implant model.

USER	TIME	Mean deviation (mm)	SD (mm)	Max + (mm)	Min- (mm)	Total deviation implant center Implant 1 (mm)	X (mm)	Y (mm)	Z (mm)	Total deviation implant center Implant 2 (mm)	X (mm)	Y (mm)	Z (mm)	Total deviation implant center Implant 3 (mm)	X (mm)	Y (mm)	Z (mm)	Total deviation implant center Implant 4 (mm)	X (mm)	Y (mm)	Z (mm)	Scanbody	Mean (mm)
1	A	-0.002	0.103	0.225	-0.224	0.222	0.109	0.183	0.063	0.107	0.059	0.088	0.013	0.134	0.071	0.112	0.013	0.216	0.092	0.195	0.009	**GROUP 1**	**0.16975**
1	B	-0.002	0.059	0.156	-0.158	0.518	0.122	0.209	0.458	0.493	0.107	0.043	0.479	0.055	0.044	0.005	0.032	0.054	0.019	0.048	0.015	**GROUP 1**	**0.28**
1	C	-0.001	0.049	0.126	-0.126	0.005	0.005	0	0	0.139	0.052	0.129	0.007	0.027	0.016	0.015	0.015	0.055	0.031	0.043	0.014	**GROUP 1**	**0.0565**
1	A	0.001	0.028	0.095	-0.082	0.086	0.049	0.067	0.02	0.022	0.014	0.005	0.016	0.025	0.014	0.018	0.009	0.047	0.017	0.036	0.024	**GROUP 2**	**0.045**
1	B	0.001	0.046	0.128	-0.119	0.111	0.036	0.088	0.056	0.073	0.024	0.047	0.05	0.048	0.004	0.03	0.037	0.072	0.031	0.034	0.055	**GROUP 2**	**0.076**
1	C	-0.007	0.097	0.199	-0.192	0.039	0.036	0.013	0.009	0.035	0.018	0.018	0.024	0.293	0.203	0.179	0.112	0.294	0.24	0.161	0.055	**GROUP 2**	**0.16525**
1	A	0.003	0.083	0.211	-0.233	0.347	0.214	0.085	0.259	0.172	0.062	0.16	0.006	0.021	0.015	0	0.015	0.106	0.026	0.102	0.003	**GROUP 3**	**0.1615**
1	B	-0.001	0.026	0.062	-0.062	0.008	0	0.008	0	0.074	0.074	0.002	0.005	0.082	0.026	0.036	0.069	0.016	0.008	0.013	0.003	**GROUP 3**	**0.045**
1	C	-0.001	0.038	0.112	-0.103	0.473	0.287	0.059	0.371	0.288	0.206	0.201	0.003	1.6	0.579	0.316	1.464	2.197	0.703	0.203	2.071	**GROUP 3**	**1.1395**
2	A	0	0.032	0.129	-0.129	0.04	0.032	0.021	0.008	0.066	0.003	0.024	0.061	0.07	0.01	0.069	0.007	0.049	0.007	0.048	0.002	**GRUPO 1**	**0.05625**
2	B	-0.005	0.092	0.206	-0.204	0.126	0.046	0.11	0.041	0.148	0.043	0.141	0.014	0.09	0.033	0.082	0.016	0.186	0.079	0.165	0.029	**GRUPO 1**	**0.1375**
2	C	-0.001	0.051	0.211	-0.21	0.055	0.036	0.037	0.017	0.19	0.069	0.002	0.177	0.026	0.021	0.012	0.006	0.025	0.01	0.017	0.015	**GRUPO 1**	**0.074**
2	A	-0.004	0.062	0.205	-0.202	0.062	0.059	0.019	0	0.088	0.065	0.059	0.009	0.048	0.035	0.03	0.01	0.083	0.069	0.043	0.013	**GRUPO 2**	**0.07025**
2	B	-0.003	0.054	0.16	-0.16	0.062	0.017	0.057	0.017	0.09	0.086	0.004	0.028	0.054	0.029	0.031	0.034	0.08	0.069	0.038	0.009	**GRUPO 2**	**0.0715**
2	C	-0.003	0.058	0.125	-0.124	0.021	0.013	0.016	0.002	0.09	0.003	0.09	0.002	0.137	0.105	0.085	0.021	0.169	0.136	0.087	0.048	**GRUPO 2**	**0.10425**
2	A	-0.002	0.041	0.1	-0.1	0.029	0.001	0.028	0.004	0.023	0.015	0.017	0	0.159	0.042	0.153	0.007	0.116	0.045	0.107	0.003	**GRUPO 3**	**0.08175**
2	B	-0.001	0.028	0.078	-0.078	0.015	0.005	0.014	0	0.018	0.007	0.016	0.001	0.113	0.063	0.071	0.06	0.021	0.003	0.02	0.001	**GRUPO 3**	**0.04175**
2	C	0.001	0.071	0.157	-0.163	0.258	0.012	0.254	0.044	0.201	0.062	0.178	0.067	0.037	0.035	0.007	0.011	0.058	0.04	0.04	0.008	**GRUPO 3**	**0.1385**
3	A	-0.005	0.097	0.259	-0.256	0.047	0.039	0.006	0.024	0.033	0.012	0.013	0.027	0.339	0.326	0.092	0.002	0.375	0.339	0.16	0.012	**GRUPO 1**	**0.1985**
3	B	-0.007	0.12	0.367	-0.365	0.042	0.031	0.022	0.018	0.068	0.023	0.062	0.012	0.249	0.155	0.194	0.016	0.34	0.25	0.229	0.011	**GRUPO 1**	**0.17475**
3	C	-0.002	0.068	0.251	-0.248	0.139	0.004	0.029	0.136	0.08	0.018	0.071	0.031	0.043	0.035	0.017	0.017	0.067	0.04	0.054	0.003	**GRUPO 1**	**0.08225**
3	A	0.001	0.053	0.149	-0.148	0.168	0.119	0.117	0.013	0.026	0.021	0.001	0.015	0.11	0.107	0.013	0.02	0.048	0.046	0.013	0.004	**GRUPO 2**	**0.088**
3	B	0.001	0.035	0.104	-0.094	0.062	0.039	0.048	0.003	0.017	0.013	0.011	0.002	0.014	0.011	0.008	0.003	0.147	0.058	0.121	0.059	**GRUPO 2**	**0.06**
3	C	0	0.024	0.079	-0.077	0.012	0.005	0.011	0.001	0.077	0.066	0.026	0.029	0.051	0.01	0.044	0.024	0.02	0.018	0.008	0.004	**GRUPO 2**	**0.04**
3	A	-0.001	0.028	0.077	-0.077	0.035	0.004	0.035	0.005	0.104	0.02	0.098	0.029	0.012	0.009	0.005	0.005	0.017	0.015	0.006	0.003	**GRUPO 3**	**0.042**
3	B	-0.002	0.113	0.3	-0.298	0.054	0.023	0.047	0.009	0.024	0.022	0.002	0.008	0.299	0.02	0.297	0.025	0.458	0.109	0.444	0.023	**GRUPO 3**	**0.20875**
3	C	-0.003	0.056	0.18	-0.178	0.21	0.03	0.201	0.053	0.157	0.017	0.154	0.023	0.027	0.002	0.026	0.005	0.023	0.021	0.009	0	**GRUPO 3**	**0.10425**
4	A	-0.013	0.168	0.341	-0.341	0.056	0.033	0.042	0.014	0.09	0.075	0.047	0.017	0.464	0.358	0.294	0.02	0.516	0.353	0.376	0.014	**GRUPO 1**	**0.2815**
4	B	0	0.044	0.126	-0.126	0.023	0.022	0.004	0.003	0.129	0.125	0.028	0.001	0.044	0.042	0.011	0.008	0.191	0.188	0.036	0.004	**GRUPO 1**	**0.09675**
4	C	-0.005	0.111	0.243	-0.243	0.419	0.045	0.396	0.129	0.328	0.083	0.316	0.034	0.023	0.01	0.009	0.018	0.048	0.037	0.029	0.009	**GRUPO 1**	**0.2045**
4	A	0.001	0.049	0.127	-0.127	0.029	0.017	0.023	0.001	0.079	0.036	0.07	0.001	0.125	0.052	0.105	0.041	0.227	0.093	0.198	0.058	**GRUPO 2**	**0.115**
4	B	-0.008	0.102	0.226	-0.221	0.027	0.026	0.006	0.007	0.059	0.045	0.038	0.006	0.379	0.307	0.051	0.215	0.313	0.304	0.048	0.059	**GRUPO 2**	**0.1945**
4	C	-0.005	0.152	0.35	-0.35	0.595	0.564	0.186	0.027	0.393	0.383	0.008	0.089	0.041	0.03	0.008	0.026	0.111	0.06	0.08	0.045	**GRUPO 2**	**0.285**
4	A	0	0.025	0.06	-0.06	0.063	0.028	0.055	0.012	0.004	0.004	0	0	0.066	0.056	0.031	0.012	0.035	0.008	0.031	0.012	**GRUPO 3**	**0.042**
4	B	-0.001	0.041	0.153	-0.118	0.142	0.111	0.087	0.014	0.03	0.011	0.028	0	0.011	0.008	0.006	0.003	0.122	0.066	0.097	0.03	**GRUPO 3**	**0.07625**
4	C	0	0.082	0.208	-0.203	0.063	0.062	0.004	0.009	0.051	0.048	0.011	0.014	0.178	0.038	0.173	0.02	0.329	0.109	0.309	0.019	**GRUPO 3**	**0.15525**
5	A	-0.007	0.166	0.396	-0.383	0.617	0.209	0.564	0.137	0.425	0.027	0.375	0.198	0.089	0.081	0.037	0.006	0.135	0.078	0.109	0.008	**GRUPO 1**	**0.3165**
5	B	-0.002	0.066	0.186	-0.185	0.033	0.003	0.03	0.013	0.181	0.062	0.164	0.046	0.045	0.028	0.019	0.03	0.027	0.001	0.018	0.019	**GRUPO 1**	**0.0715**
5	C	-0.002	0.059	0.139	-0.139	0.141	0.067	0.118	0.036	0.202	0.152	0.105	0.08	0.038	0.018	0.033	0.006	0.043	0.01	0.042	0.001	**GRUPO 1**	**0.106**
5	A	-0.006	0.089	0.207	-0.209	0.294	0.289	-0.055	0	0.299	0.283	0.089	0.031	0.023	0.022	0.005	0.004	0.03	0	0.028	0.011	**GRUPO 2**	**0.1615**
5	B	0	0.032	0.12	-0.119	0.061	0.012	0.046	0.036	0.111	0.084	0.073	0.004	0.014	0.011	0.008	0.004	0.016	0.002	0.016	0.004	**GRUPO 2**	**0.0505**
5	C	-0.001	0.041	0.172	-0.171	0.049	0.029	0.037	0.012	0.099	0.058	0.019	0.077	0.01	0.008	0.005	0.003	0.018	0.012	0.012	0.004	**GRUPO 2**	**0.044**
5	A	0	0.084	0.218	-0.213	0.144	0.083	0.113	0.032	0.021	0.02	0.005	0.003	0.23	0.124	0.194	0.001	0.24	0.02	0.238	0.019	**GRUPO 3**	**0.15875**
5	B	-0.001	0.03	0.091	-0.09	0.052	0.014	0.04	0.03	0.106	0.052	0.091	0.013	0.016	0.006	0.008	0.012	0.02	0.007	0.015	0.011	**GRUPO 3**	**0.0485**
5	C	0.001	0.031	0.089	-0.097	0.079	0.005	0.063	0.048	0.023	0.008	0.021	0.001	0.057	0.012	0.055	0.001	0.081	0.056	0.057	0.003	**GRUPO 3**	**0.06**

### 2-Implant model

**Fig. 13:**
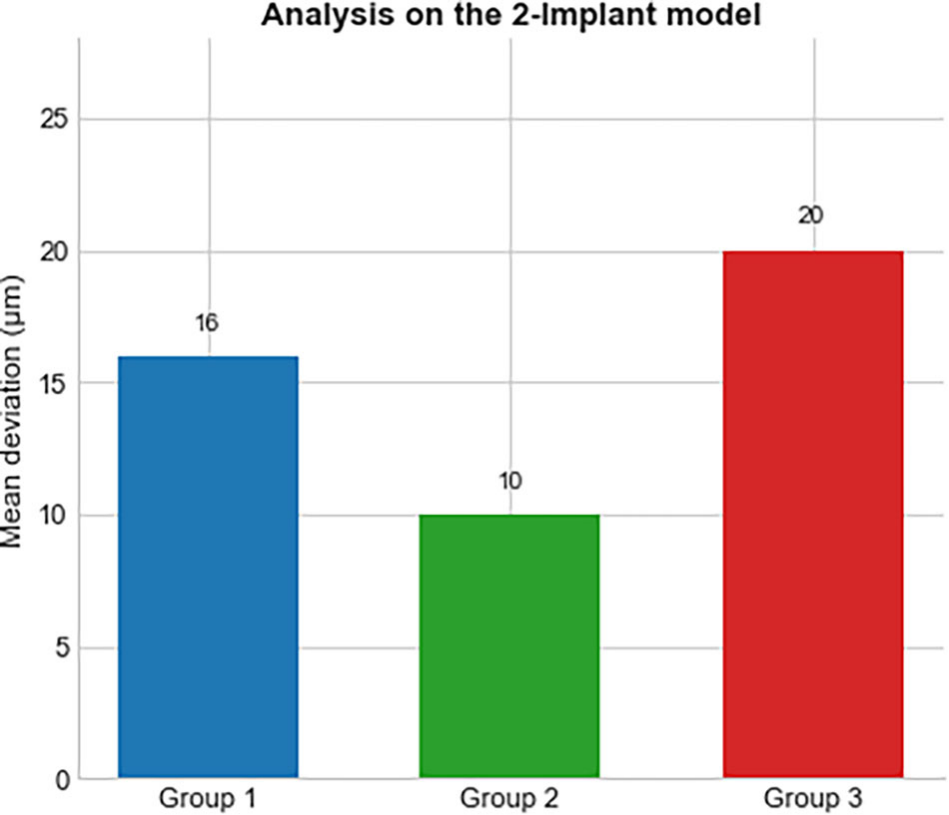
Mean deviation (µm) comparison between Group 1, Group 2, and Group 3 in the 2-implant model. The graph shows the average deviations measured for each scan body group. Group 1-16 microns, Group 2-10 microns and Group 3-20 microns.

The 2-implant model demonstrated the lowest deviations overall (Fig. 13, Table 9).

- Group 1: Mean deviation 16 μm.
- Group 2: Mean deviation 10 μm, the best overall performance.
- Group 3: Mean deviation 20 μm, without advantage over two flat surfaces.

**Table 9:** Results of the analysis on the 2-implant model.

USER	TIME	Mean deviation (mm)	SD (mm)	Max + (mm)	Min- (mm)	Total deviation implant center Implant 1 (mm)	X (mm)	Y (mm)	Z (mm)	Total deviation implant center Implant 2 (mm)	X (mm)	Y (mm)	Z (mm)	Scanbody	Mean (mm)
1	A	−0.001	0.047	0.142	−0.142	0.027	0.001	0.025	0.011	0.05	0.003	0.049	0.011	**GRUPO 1**	**0.0385**
1	B	0	0.028	0.075	−0.054	0.036	0.003	0.036	−0.001	0.034	0.003	0.034	0.034	**GRUPO 1**	**0.035**
1	C	0	0.033	0.076	−0.076	0.047	0.005	0.047	0.002	0.053	0.005	0.052	0.003	**GRUPO 1**	**0.05**
1	A	0	0.012	0.045	−0.044	0.011	0	0.011	0.003	0.004	0.002	0.002	0.002	**GRUPO 2**	**0.0075**
1	B	0	0.014	0.057	−0.057	0.008	0.001	0.007	0.004	0.004	0.002	0.002	0.003	**GRUPO 2**	**0.006**
1	C	0	0.017	0.062	−0.061	0.017	0.003	0.016	0.004	0.006	0.002	0.004	0.004	**GRUPO 2**	**0.0115**
1	A	0	0.007	0.028	−0.028	0.001	0	0	0	0.004	0.002	0.003	0	**GRUPO 3**	**0.0025**
1	B	0	0.009	0.033	−0.033	0.005	0.001	0.004	0.002	0.004	0.001	0.002	0.002	**GRUPO 3**	**0.0045**
1	C	0	0.01	0.03	−0.03	0.009	0.004	0.008	0.001	0.015	0.005	0.014	0.001	**GRUPO 3**	**0.012**
2	A	0	0.008	0.024	−0.023	0.008	0	0.008	0	0.007	0	0.007	0	**GRUPO 1**	**0.0075**
2	B	0	0.018	0.054	−0.054	0.006	0.002	0.003	0.004	0.014	0	0.013	0.004	**GRUPO 1**	**0.01**
2	C	0	0.016	0.051	−0.051	0.006	0.002	0.004	0.004	0.012	0	0.012	0.004	**GRUPO 1**	**0.009**
2	A	0	0.014	0.058	−0.056	0.006	0.001	0.005	0.003	0.005	0.001	0.004	0.002	**GRUPO 2**	**0.0055**
2	B	0	0.01	0.041	−0.04	0.004	0.002	0.001	0.002	0.009	0.001	0.009	0.002	**GRUPO 2**	**0.0065**
2	C	0	0.012	0.042	−0.04	0.004	0.002	0	0.003	0.011	0.014	0.01	0.003	**GRUPO 2**	**0.0075**
2	A	0	0.007	0.024	−0.024	0.004	0.001	0.003	0	0.005	0.001	0.005	0	**GRUPO 3**	**0.0045**
2	B	0	0.004	0.011	−0.011	0.007	0	0.006	0	0.008	0.001	0.008	0	**GRUPO 3**	**0.0075**
2	C	0	0.004	0.017	−0.017	0.004	0	0.003	0.001	0.002	0.001	0.002	0.001	**GRUPO 3**	**0.003**
3	A	0	0.008	0.028	−0.029	0.005	0.001	0.004	0.002	0.003	0.002	0	0.002	**GRUPO 1**	**0.004**
3	B	0	0.018	0.059	−0.088	0.008	0.001	0.005	0.007	0.01	0	0.007	0.007	**GRUPO 1**	**0.009**
3	C	0	0.02	0.069	−0.095	0.008	0.002	0.003	0.006	0.01	0.001	0.008	0.006	**GRUPO 1**	**0.009**
3	A	0	0.009	0.038	−0.037	0.002	0.001	0	0.002	0.004	0.001	0.004	0.001	**GRUPO 2**	**0.003**
3	B	0	0.005	0.02	−0.02	0.002	0	0.001	0	0.004	0.001	0.004	0	**GRUPO 2**	**0.003**
3	C	0	0.005	0.021	−0.02	0.003	0	0.003	0	0.001	0.001	0	0.001	**GRUPO 2**	**0.002**
3	A	0	0.005	0.014	−0.014	0.01	0.002	0.01	0	0.01	0.001	0.01	0	**GRUPO 3**	**0.01**
3	B	0	0.009	0.033	−0.03	0.006	0	0.006	0.003	0.004	0.001	0.003	0.002	**GRUPO 3**	**0.005**
3	C	0	0.008	0.03	−0.029	0.01	0.001	0.01	0	0.008	0.003	0.008	0.001	**GRUPO 3**	**0.009**
4	A	0	0.006	0.018	−0.018	0.005	0.001	0.005	0	0.007	0.001	0.007	0	**GRUPO 1**	**0.006**
4	B	0	0.024	0.078	−0.069	0.03	0.002	0.03	0.005	0.022	0	0.022	0.005	**GRUPO 1**	**0.026**
4	C	0	0.018	0.048	−0.048	0.017	0	0.016	0.005	0.027	0.002	0.026	0.005	**GRUPO 1**	**0.022**
4	A	0	0.007	0.017	−0.019	0.011	0.005	0.001	0	0.013	0.006	0.012	0	**GRUPO 2**	**0.012**
4	B	0	0.026	0.049	−0.049	0.041	0.015	0.038	0.001	0.041	0.014	0.038	0.001	**GRUPO 2**	**0.041**
4	C	0	0.005	0.015	−0.015	0.003	0.003	0.001	0	0.003	0.003	0	0	**GRUPO 2**	**0.003**
4	A	0	0.035	0.067	−0.067	0.058	0	0.058	0.002	0.058	0.001	0.058	0.003	**GRUPO 3**	**0.058**
4	B	−0.001	0.039	0.082	−0.082	0.065	0	0.065	0.003	0.062	0	0.062	0.004	**GRUPO 3**	**0.0635**
4	C	0	0.031	0.058	−0.058	0.054	0	0.054	0	0.057	0.003	0.057	0.001	**GRUPO 3**	**0.0555**
5	A	0	0.017	0.053	−0.077	0.008	0.001	0.003	0.006	0.01	0	0.008	0.006	**GRUPO 1**	**0.009**
5	B	0	0.009	0.026	−0.026	0.004	0.002	0.001	0.003	0.005	0	0.004	0.003	**GRUPO 1**	**0.0045**
5	C	0	0.026	0.077	−0.095	0.012	0	0.009	0.009	0.013	0.003	0.008	0.01	**GRUPO 1**	**0.0125**
5	A	0	0.016	0.044	−0.044	0.02	0	0.02	0.001	0.025	0.002	0.025	0.001	**GRUPO 2**	**0.0225**
5	B	0	0.015	0.047	−0.047	0.014	0.002	0.014	0.002	0.021	0.002	0.021	0.002	**GRUPO 2**	**0.0175**
5	C	0	0.011	0.035	−0.035	0.009	0.002	0.009	0.002	0.017	0.005	0.016	0.002	**GRUPO 2**	**0.013**
5	A	0	0.018	0.044	−0.044	0.025	0.003	0.025	0.001	0.03	0.003	0.03	0	**GRUPO 3**	**0.0275**
5	B	0	0.018	0.046	−0.046	0.018	0.003	0.02	0	0.02	0.002	0.02	0	**GRUPO 3**	**0.019**
5	C	0	0.02	0.058	−0.06	0.005	0	0.004	0.002	0.057	0.004	0.056	0.008	**GRUPO 3**	**0.031**

ANOVA revealed a significant effect of implant number on trueness (*F* = 15.97, *p* < 0.001), with Tukey’s post-hoc test confirming that the 6-implant model exhibited significantly greater deviations than both the 2- and 4-implant models (*p* < 0.001), while no difference was found between the 2- and 4-implant configurations. In contrast, scan body design did not show a statistically significant effect across models (p > 0.05), although mean deviations demonstrated a consistent trend: the reduced-length design (Group 2) yielded lower deviations (87 μm in the 6-implant model; 104 μm in the 4-implant model; 10 μm in the 2-implant model) compared with the standard (Group 1) and three-flat-surface designs (Group 3). Operator influence was generally not significant, except in the 4-implant model, where performance varied between examiners (*F* = 5.62, *p* = 0.001). So, the null hypothesis could not be rejected.

## Discussion

This study evaluated how scan body characteristics—specifically total length and head geometry influence scanning trueness across models with varying implant configurations. The findings demonstrate that small modifications in scan body design can result in substantial differences in the accuracy of digital impressions. Notably, a reduction in scan body length was associated with improved trueness, particularly in controlled settings with multiple implants.

Digital implant impressions are increasingly adopted by clinicians as a replacement for conventional analogue impressions, due to their promising accuracy [16]. However, the existing scientific evidence remains inconclusive regarding the optimal design for intraoral scan bodies [17–20]. Thus, a wide variability of designs is currently available. A recent systematic review [21] evaluated the impact of scan body design on digital accuracy—focusing on height, diameter, geometry, material, and retention system but concluded that no definitive evidence supports a single optimal design.

The scan body height has been repeatedly identified as critical for the trueness of digital impressions, particularly in complex cases like complete edentulism. In line with our findings, reducing length improved trueness, which may enhance the accuracy of full-arch digital implant impressions an application where digital impressions still present clinical challenges [22].

However, shorter scan bodies introduce clinical limitations: restricted interarch space, soft tissue interference, or deep implant placement can compromise capture and seating accuracy. Although the results should be interpreted with caution when extrapolating to clinical settings, several factors could modify the observed trends in practice. For example, reduced scan body height may complicate scanning in patients with deep implant placement or limited interarch space, where visibility and accessibility are compromised. Similarly, geometric complexity may be more problematic in anatomies with divergent implants, restricted soft tissue corridors, or the presence of saliva and patient movement—all of which could introduce artefacts not captured in vitro. Thus, while reduced length appears advantageous in vitro, its clinical applicability must be carefully balanced with anatomical and procedural constraints.

The addition of a third flat surface, although intended to enhance angular registration, instead led to increased deviations in some models. This suggests that greater geometric complexity may destabilize scanning accuracy, especially in multi-implant scenarios.

The results indicate that simpler, shorter designs reduce deflection and enhance stability. Group 2 (reduced-length) consistently outperformed the standard design (Group 1), whereas Group 3 (third flat surface) increased deviations. These findings highlight that design simplification and physical stability are more effective than added complexity in optimizing digital accuracy.

All scans were performed under controlled irradiance (180–250 W/m²). Unlike most studies reporting illuminance (lux), irradiance was chosen because it accounts for the entire radiative energy spectrum, including non-visible wavelengths, offering a more comprehensive control of environmental conditions [6].

A limitation of this study is the restricted material selection. Although titanium was used for precision, polymer and hybrid scan bodies may behave differently. Based on extensive internal R&D, we observed that polymer-only scan bodies deform at the screw seat after repeated torque, falsifying implant position. Hybrid designs (polymer with titanium base) reduce this risk but introduce assembly inaccuracies during embedding, potentially leading to positional errors. By manufacturing the scan body entirely in one material (titanium), such risks are minimized, improving positional stability.

Another limitation is the in vitro setup, which cannot fully replicate intraoral conditions (soft tissue, saliva, patient movement). Consequently, while the results are reproducible, they must be interpreted cautiously. Future studies should not only aim to validate the in vitro results under real clinical conditions, but also investigate targeted aspects derived directly from this study: (1) balancing scan body height reduction with accessibility in cases of limited interarch space or deep implant placement, (2) testing simplified vs. complex geometries under real intraoral variability, (3) comparing different materials including polymer and hybrid alternatives to titanium, and (4) analyzing scanner-software interactions and environmental parameters such as irradiance stability. These focused directions are essential to translate in vitro findings into clinically applicable protocols.

Despite these limitations, this study provides valuable insights for scan body optimization. Specifically: reduced length improved trueness; additional flat surfaces increased deviations; and design simplification and stability appear key for digital accuracy.

Future research should assess not only geometry and length but also platform switching, surface treatments, and scanner-software interactions, as well as environmental parameters (irradiance variation, equipment stability). Formal statistical analysis confirmed that the number of implants had a predominant effect on impression trueness, with the 6-implant configuration showing significantly greater deviations than the 2 and 4 implant models. Although scan body geometry did not reach statistical significance, the descriptive results consistently indicated improved accuracy with the reduced-length design (Group 2) and greater deviations with the three-flat-surface design (Group 3). This apparent discrepancy between descriptive trends and statistical outcomes may be attributed to the modest effect size of scan body design relative to implant number, as well as the limited number of repetitions per condition, which constrained statistical power. Despite the lack of significance, the consistent trend toward better performance of the reduced-length design suggests that scan body geometry may still play a clinically relevant role in optimizing digital impression accuracy.

In summary, this study underscores the critical role of scan body design and standardized scanning conditions in achieving high trueness within digital implant workflows. By systematically evaluating how specific design elements—such as length and head geometry—affect scanning accuracy, the findings offer valuable insights for optimizing both clinical protocols and product development. A deeper understanding of these variables enables clinicians and manufacturers to enhance the reliability, precision, and predictability of digital dentistry procedures.

## Conclusions

This study describes how scan body geometry shows a tendency to impact the trueness of intraoral digital implant impressions. Among the three designs tested, Group 2 (featuring a 50% reduction in height) consistently yielded the lowest deviation values across all tested in vitro models. Specifically, in the short-span bridge case, Group 2 achieved a mean deviation of 10 μm, compared to 16 μm in Group 1 and 20 μm in Group 3. Similarly, in the 4-implants model, deviations were 104 μm, 153 μm, and 166 μm for Groups 2, 1, and 3, respectively. In the fully edentulous case, Group 2 again outperformed the others, with a deviation of 87 μm, followed by Group 1 at 102 μm and Group 3 at 149 μm. These results suggest that reducing the height of the scan body enhances scan accuracy, likely by improving scanner accessibility and reducing potential distortion. On the other hand, the addition of a third flat surface in Group 3 increased deviation, introducing angular inconsistencies in some models. However, this observation is limited to the laboratory setting and requires further clinical validation. While limited to in vitro conditions, these findings highlight the importance of optimizing scan body geometry to improve accuracy in digital implant workflows.

## Data Availability

The datasets used and/or analyzed during the current study are available from the corresponding author on reasonable request.
